# Support ecology in master’s research development: perceived supervisor guidance and graduate affairs management support

**DOI:** 10.3389/fpsyg.2026.1927035

**Published:** 2026-10-20

**Authors:** Min Zhu, Siyu Zhou

**Affiliations:** 1 School of Computer Science and Technology, Zhejiang Sci-Tech University, Hangzhou, China; 2 School of Public Administration, Hangzhou Normal University, Hangzhou, China

**Keywords:** graduate affairs management support, graduate students, latent profile analysis, loose coupling theory, perceived supervisor guidance, research development, research engagement, support ecology

## Abstract

**Objective:**

Graduate students’ research development is shaped not only by academic guidance but also by how students perceive the broader institutional support environment in which research training occurs. This study examined the associations of perceived supervisor guidance and perceived graduate affairs management support with master’s students’ self-reported research development status and explored heterogeneous configurations of these two forms of support.

**Methods:**

A convergent mixed-methods design with quantitative priority was adopted. Survey data were collected from 378 full-time master’s students at three science and engineering universities in Zhejiang Province, China. Analyses included regression models with university fixed effects, bootstrap indirect-association analysis, interaction analysis, latent profile analysis, and BCH-adjusted distal-outcome comparisons. Concurrent semi-structured interviews with 18 graduate students, five supervisors, and six graduate affairs administrators were analyzed thematically to contextualize the quantitative findings.

**Results:**

When both support variables were included simultaneously, perceived supervisor guidance (*B* = 0.165, *p* = 0.003) and perceived graduate affairs management support (*B* = 0.364, *p* < 0.001) were independently and positively associated with self-reported research development status. A significant cross-sectional indirect association was observed through perceived graduate affairs management support (*B* = 0.156, bootstrap 95% CI [0.103, 0.217]), whereas the interaction between the two forms of support was not significant (*B* = 0.005, *p* = 0.933). Four support profiles were identified: fully supported (34.1%), management-support-dominant (24.9%), supervisor-support-dominant (27.2%), and insufficiently supported (13.8%). Qualitative findings further indicated that supervisor guidance primarily addressed substantive academic and research-task needs, whereas graduate affairs management support primarily provided procedural, informational, developmental, and risk-related support and could not replace academic supervision.

**Conclusion:**

Master’s research development is embedded in a support ecology in which perceived academic and institutional support represent distinct but interconnected contextual resources. By extending the conventional supervisor–student dyadic perspective toward a multi-actor support ecology, this study provides a basis for strengthening supervisor guidance, improving graduate affairs management support, and developing differentiated support strategies for graduate research development.

## 1 Introduction

Master’s students’ research development is an important dimension of graduate education quality. It is reflected not only in research outputs, but also in students’ sustained engagement in research, the development of research competence, their understanding of academic norms, and their ability to maintain a relative balance between research pressure and personal life (Yusuf et al., 2020). This issue is particularly salient in science and engineering universities. Research training for master’s students in these contexts usually depends on supervisor guidance, research group operation, team collaboration, and practice-based research settings. Students’ research development is therefore shaped through the interaction of project tasks, supervisor guidance, and institutional training arrangements (Hammond, 2021). Accordingly, the research development of master’s students in science and engineering universities is often characterized by stronger supervisor dependence, organizational embeddedness, and process uncertainty.

From an educational psychology perspective, graduate research development can also be understood as a learning and developmental process shaped partly by how students perceive the support available in their research environment. Perceived support may provide contextual resources for sustained research engagement, competence development, persistence when research difficulties arise, and the management of research-related demands. Previous studies have linked graduate students’ perceived supervisor support with research-related self-efficacy, persistence, innovation, and research productivity, suggesting that supervisory support has psychological and learning-related relevance in addition to its academic function (Han J. et al., 2022; Aldhi et al., 2024). In the present study, this developmental process is examined through students’ self-reported research engagement, perceived research competence, research output, and research–life balance. Research self-efficacy, motivation, and related psychological constructs are not directly measured and are therefore treated as adjacent explanatory processes rather than empirically tested mechanisms.

Existing research has widely emphasized the central role of supervisor guidance in graduate education. Through support in topic selection, research design, methodological training, thesis writing, and academic norm formation, supervisors help students enter research practice and develop research competence (Grohnert et al., 2024; Chugh et al., 2022). In science and engineering universities, the importance of supervisor guidance is particularly salient. Because students’ research activities are often closely linked to research group resources, experimental platforms, project schedules, and team collaboration, supervisors function not only as academic mentors but also as key connectors through which students enter research teams, understand project tasks, and access research opportunities. Accordingly, supervisor guidance is closely associated with graduate students’ research development (Ringo, 2025). However, explaining the research development of master’s students in science and engineering universities solely through the dyadic “supervisor–student” relationship has analytical limitations. Whether supervisor guidance can support students’ stable research progress is also related to training systems, academic process management, daily affairs support, responses to psychological pressure, and organizational coordination mechanisms when supervisor–student communication or research progress encounters difficulties (Schneider et al., 2024). This issue is especially important in project-based, team-based, and experiment-intensive science and engineering training contexts, where students’ research tasks are often intertwined with project cycles, experimental outcomes, thesis requirements, competition-based practice, and graduation review procedures (Hammond, 2021). When supervisor guidance, management systems, and graduate affairs management support are not effectively connected, students may face delayed research progress, accumulated pressure, communication imbalance, and developmental risks (Wanyama and Eyamu, 2021).

Graduate affairs management support represents a form of institutional support within the graduate training environment that is distinct from supervisors’ substantive academic guidance. In this study, it refers to students’ perceived support related to institutional implementation, academic process management, life-related services, and psychological care, including milestone reminders, training procedures, academic early warning, resource coordination, and access to relevant support services. It is therefore narrower than general organizational or student-affairs support and does not include substantive academic judgment or discipline-specific research guidance.

Loose coupling theory provides an organizational lens for understanding how supervisor guidance and graduate affairs management support may coexist within graduate training (Maassen and Stensaker, 2019). Supervisors retain substantial academic autonomy, graduate affairs administrators undertake institutional implementation and process support, and students navigate both research tasks and formal training requirements. These actors and support processes are functionally differentiated but interdependent rather than fully integrated. Accordingly, this study treats perceived supervisor guidance and perceived graduate affairs management support as distinct but potentially interconnected contextual resources within graduate students’ research-development environment. Accordingly, perceived graduate affairs management support may be associated with self-reported research development status and may form an indirect association between perceived supervisor guidance and self-reported research development status.

Existing research has examined supervisor guidance, graduate student development, and organizational support in higher education from different perspectives, yet two gaps remain particularly relevant. First, graduate research development has often been examined primarily through the supervisor–student dyad, with less attention to students’ perceptions of institutional support embedded in graduate training processes (Mason et al., 2021; Lategan, 2025). Second, relatively little is known about how perceived supervisor guidance and perceived graduate affairs management support are related to one another, whether they retain independent associations with students’ research development, and whether students experience heterogeneous combinations of these two support sources (Rönkkönen et al., 2024; Corcelles-Seuba et al., 2023). Addressing these questions can extend research on graduate supervision toward a broader support-ecology perspective while retaining attention to students’ learning and developmental experiences.

To address these gaps, this study examines the relationships among perceived supervisor guidance, perceived graduate affairs management support, and self-reported research development status among master’s students in science and engineering universities. Specifically, the study examines the direct associations of the two forms of perceived support with research development, evaluates whether a statistically significant indirect association is observed through perceived graduate affairs management support, and uses latent profile analysis to identify heterogeneous support types based on the two support domains. Concurrent qualitative interviews with graduate students, supervisors, and graduate affairs administrators are used to contextualize the quantitative patterns and clarify the differentiated functions, connections, and boundaries of academic and institutional support.

This study makes three contributions. First, theoretically, it extends the conventional supervisor–student dyadic perspective toward a multi-actor support ecology, treating students’ perceptions of academic and institutional support as differentiated but interconnected contextual resources for graduate research learning and development. Second, methodologically, it combines variable-centered and person-centered analyses with qualitative contextualization, thereby examining both overall associations and heterogeneity in students’ support experiences. Third, practically, the findings can inform more differentiated support arrangements that strengthen supervisor guidance while improving institutional processes, risk identification, and access to appropriate student support.

## 2 Materials and methods

### 2.1 Hypotheses and research questions

Based on research on supervisor–graduate student relationships and loose coupling theory, perceived supervisor guidance is expected to be positively associated with master’s students’ self-reported research development status. Supervisor guidance strengthens the academic coupling between students and research practice through support in topic selection, methodological training, thesis guidance, research norms, and resource provision (Janssen et al., 2021). For master’s students in science and engineering universities, supervisor guidance also involves helping students understand research tasks, access research resources, and produce research outcomes (Wu et al., 2024). Therefore, high-quality supervisor guidance is more likely to be associated with stronger research engagement, higher research competence, greater research output, and better research–life balance. Previous educational-psychology research has similarly linked perceived supervisor support with research self-efficacy and research-related behavior among graduate students (Wang et al., 2022). Accordingly, the following hypothesis is proposed:

H1: Perceived supervisor guidance is positively associated with master’s students’ self-reported research development status.

Perceived graduate affairs management support may also be positively associated with self-reported research development status. Research on graduate affairs management support suggests that institutional clarity, academic process management, life-related support, and psychological care can reduce uncertainty in students’ developmental processes and increase the likelihood of sustained engagement in learning and research (El Alfy and Abukari, 2020). In science and engineering universities, such support may bridge insufficient connections between everyday research practice and formal training systems, helping students understand academic milestones, buffer developmental pressure, and maintain research engagement. Accordingly, the following hypothesis is proposed:

H2: Perceived graduate affairs management support is positively associated with master’s students’ self-reported research development status.

Perceived supervisor guidance may also be positively associated with perceived graduate affairs management support. Effective supervisor guidance is often embedded in a training system in which supervisors interact with graduate affairs management support. Such interaction may contribute to the quality of graduate training and reflect a higher level of collaborative coupling within the training system (Klein, 2020). Accordingly, the following hypothesis is proposed:

H3: Perceived supervisor guidance is positively associated with perceived graduate affairs management support.

The positive association between perceived supervisor guidance and self-reported research development status may coexist with an indirect pattern involving perceived graduate affairs management support. Students who report more favorable supervisor guidance may also report more favorable institutional support conditions, while perceived graduate affairs management support may in turn be associated with their research-development experiences (Merga and Mason, 2021). Within a loosely coupled training environment, such a pattern would be consistent with the possibility that academic and institutional support are interconnected, although cross-sectional data cannot establish their temporal ordering. Accordingly, the following hypothesis is proposed:

H4: Perceived supervisor guidance shows a positive indirect association with self-reported research development status through perceived graduate affairs management support.

Based on loose coupling theory, different students may experience different combinations of perceived supervisor guidance and perceived graduate affairs management support, suggesting that multiple support types may exist in theory. Therefore, this study identifies support types based on perceived supervisor guidance and perceived graduate affairs management support. The exploratory research questions are as follows:

Q1: Can distinct support types be identified among master’s students based on perceived supervisor guidance and perceived graduate affairs management support?

Q2: Do master’s students in different support types differ in self-reported research development status?

Q3: How do graduate students, supervisors, and graduate affairs administrators describe the differentiated functions, connections, and boundaries of supervisor guidance and graduate affairs management support in relation to graduate students’ research development?

In summary, this study examines the associations among perceived supervisor guidance, perceived graduate affairs management support, and self-reported research development status. It then identifies heterogeneous support types and uses qualitative evidence to contextualize the differentiated functions, connections, and boundaries of the two support domains. In doing so, the study examines how supervisor guidance, institutional support, and graduate students’ research development are connected within the loosely coupled training system of science and engineering universities (Figure 1).

**FIGURE 1:**
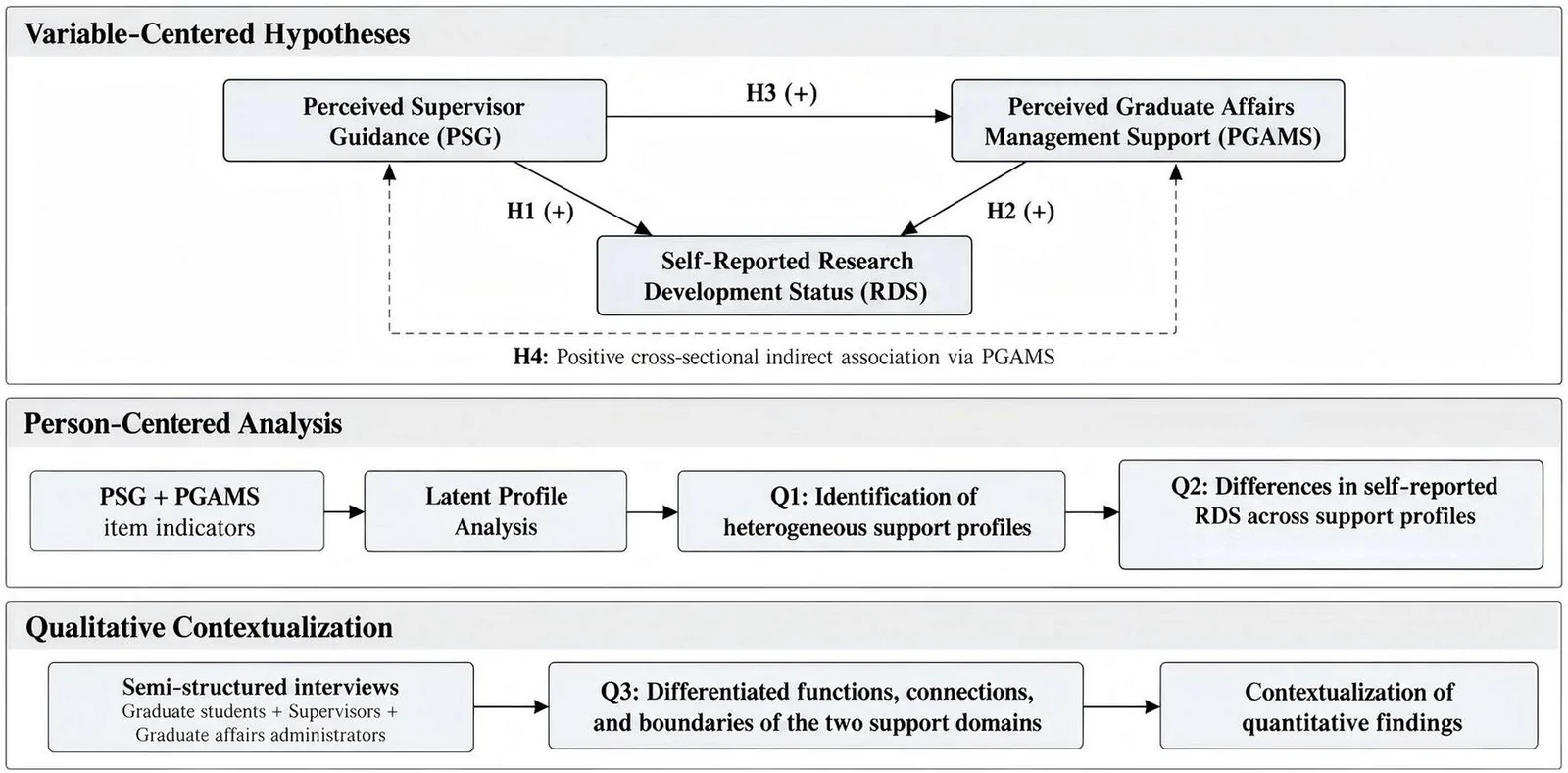
Conceptual framework, hypotheses, and research questions.

### 2.2 Research design

This study adopted a convergent mixed-methods design with quantitative priority (QUAN + qual) to examine perceived supervisor guidance, perceived graduate affairs management support, and self-reported research development status among master’s students in science and engineering universities. The quantitative survey and qualitative interviews were conducted during the same data-collection period. The quantitative component was given priority and was used to examine associations among the focal constructs and heterogeneous configurations of perceived support, whereas the qualitative component provided contextual accounts of how academic and institutional support were experienced in graduate training.

For the quantitative component, the study first used survey data to examine the relationships among perceived supervisor guidance, perceived graduate affairs management support, and self-reported research development status (RDS). Descriptive statistics and Pearson correlation analysis were used to characterize the focal variables and examine their bivariate associations, followed by multiple linear regression to test the independent associations of perceived supervisor guidance and perceived graduate affairs management support with RDS. A bootstrap indirect-association analysis was then conducted to examine whether perceived supervisor guidance showed a statistically significant indirect association with RDS through perceived graduate affairs management support. An additional interaction analysis examined whether the association between perceived supervisor guidance and RDS varied according to the level of perceived graduate affairs management support. The person-centered analysis then used latent profile analysis to identify heterogeneous support types based on the item-level indicators of the two support domains, followed by BCH-adjusted distal-outcome analysis to compare RDS across the identified profiles while accounting for classification uncertainty. For the qualitative component, semi-structured interview data from graduate students, supervisors, and graduate affairs administrators were analyzed thematically and integrated with the quantitative findings to contextualize the differentiated functions, connections, and boundaries of academic and institutional support in graduate research training.

### 2.3 Quantitative survey: participants and procedure

This study used a two-stage sampling procedure and conducted a cross-sectional questionnaire survey from March to June 2023 in three science and engineering universities in Zhejiang Province, China. The selection of these three universities was based on the following considerations. First, all three universities had a relatively stable scale of master’s student training in science and engineering, which provided a sufficient pool of potential participants for this study. Second, all three universities regarded graduate affairs management support as an important measure in graduate training. Third, the three universities were located in the same province and shared a relatively similar regional higher education policy environment, which helped reduce the potential influence of regional institutional differences on the study results.

At the student sampling stage, stratified random sampling was used. The participants were full-time master’s students in science and engineering disciplines at the three universities, including first-year, second-year, and third-year master’s students. The sample size was estimated using a commonly used formula for cross-sectional surveys: $n=\frac{{\text{Z}}^{2}\,p\,\left(1-p\right)}{{\text{d}}^{2}}$. Under a 95% confidence level and a 5% margin of error, the theoretical minimum sample size was 384. Considering that the target population of this study consisted of full-time master’s students in science and engineering disciplines at three science and engineering universities in Zhejiang Province, with an estimated population size of approximately 12,000, the finite population correction formula was further applied:$n=\frac{{\text{Nn}}_{0}}{\text{N}+{\text{n}}_{0}-1}$. The minimum required sample size was 372. To ensure balanced representation across universities and grade levels, university and grade were used as stratification criteria. Within each of the nine university-by-grade strata, 50 students were randomly selected from the corresponding graduate student roster, resulting in 450 questionnaires being distributed. A total of 378 valid questionnaires were obtained, yielding an overall valid response rate of 84.0% and exceeding the minimum required sample size after finite population correction (Table 1). The final sample included 141 first-year, 119 second-year, and 118 third-year master’s students.

**TABLE 1:** Demographic characteristics of the quantitative survey sample (*N* = 378).

Variable	Category	*n*	%
Gender	Male	255	67.5
	Female	123	32.5
Grade	First-year master’s student	141	37.3
	Second-year master’s student	119	31.5
	Third-year master’s student	118	31.2
Supervisor academic title	Professor	204	54.0
	Associate professor	148	39.2
	Lecturer	26	6.9
Supervisor age	Under 30 years	5	1.3
	30–39 years	121	32.0
	40–49 years	165	43.7
	50–59 years	80	21.2
	60 years and above	7	1.9
Number of master’s students supervised by the supervisor	5 or fewer	138	36.5
	More than 5	240	63.5

The inclusion criteria were as follows: (1) full-time master’s students in science and engineering disciplines at the participating universities; (2) students who were able to independently understand and complete the questionnaire; and (3) students who provided informed consent to participate. During data cleaning, duplicate submissions and questionnaires with substantial missing data, obvious logical inconsistencies, or highly patterned responses were excluded.

The survey followed the principles of voluntary participation, confidential completion, and the right to withdraw at any time. Before the formal survey, all participants were informed of the study purpose, data use, and confidentiality arrangements, and they completed the questionnaire on the basis of informed consent. Only participants who actively indicated electronic informed consent were able to proceed to the online questionnaire.

### 2.4 Measures

The questionnaire contained 13 positively worded items assessing three student-reported constructs: perceived supervisor guidance (PSG; five items), perceived graduate affairs management support (PGAMS; four items), and self-reported research development status (RDS; four items). For PSG and PGAMS, students evaluated their satisfaction using a five-point scale ranging from 1 (very dissatisfied) to 5 (very satisfied). For RDS, students rated their agreement with statements about their current research-development status from 1 (strongly disagree) to 5 (strongly agree). Mean item scores were calculated for each construct, with higher values representing more positive perceived support or research-development status. For latent profile analysis, the nine item-level PSG and PGAMS scores were used as profile indicators (Table 2).

**TABLE 2:** Reliability and construct validity of the three study measures.

Analysis type	Indicator	Result
Internal consistency	Overall Cronbach’s alpha	0.866
	Supervisor guidance	0.850
	Graduate affairs management support	0.841
	Research development status	0.843
Construct validity	KMO value	0.885
	Bartlett’s test χ^2^	2066.899
	Bartlett’s test *p*-value	< 0.001
Principal component analysis	Number of retained components	3
	Cumulative variance explained	66.04%
	Range of primary component loadings	0.745–0.829

The questionnaire was adapted and integrated from prior research rather than reproduced directly from a single standardized instrument. The supervisor-guidance items were informed particularly by research on academic, personal, and autonomy support in graduate supervision (Overall et al., 2011), and were contextualized with reference to Chinese studies of supervisor–graduate student relationships and supervisory practices (Wang et al., 2018; Han X. et al., 2022). The PGAMS and RDS items were developed by integrating relevant graduate-education literature with the institutional and developmental contexts addressed in the present study. The preliminary questionnaire was reviewed by five experts in higher education, and a pilot test was conducted with 50 master’s students before the formal survey. The formal survey instrument was administered entirely in Chinese. No English version was used for data collection; English translations of the original Chinese items are provided only for reporting purposes. The complete Chinese item wording and corresponding English translations are presented in Supplementary Table 1.

#### 2.4.1 Perceived supervisor guidance

Perceived supervisor guidance (PSG) was assessed using five items covering five aspects of students’ supervisory experiences: academic guidance competence, guidance style, supervisory philosophy, timeliness of guidance, and resource provision. The items asked students to evaluate their satisfaction with their supervisor’s ability to clarify research directions and ideas, use appropriate and individualized guidance approaches, foster independent research ability and academic norms, provide timely feedback when research problems arose, and provide access to resources such as funding, equipment, data, platforms, and academic exchange opportunities. The mean of the five items was used as the PSG score, with higher scores representing more positive perceptions of supervisor guidance.

#### 2.4.2 Perceived graduate affairs management support

Perceived graduate affairs management support (PGAMS) was assessed using four items covering students’ satisfaction with institutional implementation, academic process management, life-related support, and psychological care provided by their college, university, or graduate management/service units. The items addressed implementation of graduate-training regulations, clarity and service in academic administrative procedures, life-related support such as accommodation, financial assistance, medical services, and learning conditions, and attention to graduate students’ psychological state and related support. In this study, PGAMS therefore refers specifically to students’ perceptions of institutional and process-based support surrounding graduate training. It does not encompass supervisors’ substantive academic judgment, discipline-specific research guidance, or direct responsibility for students’ research projects. The mean of the four items was used as the PGAMS score, with higher scores indicating more favorable perceived support.

#### 2.4.3 Self-reported research development status

Self-reported research development status (RDS) was assessed using four items covering research engagement, perceived research competence, research output, and research–life balance. Students indicated the extent to which they were currently actively engaged in research, believed they possessed the skills needed to conduct research in their discipline, had achieved research outputs such as papers, patents, projects, reports, or competition outcomes, and were able to balance research activities with personal life while maintaining psychological and physical well-being. The mean of the four items was used as the RDS score, with higher scores indicating more favorable self-reported research development status. The measure therefore reflects students’ own evaluation of their current research-development status.

#### 2.4.4 Control variables

The control variables in this study included graduate students’ gender, graduate students’ grade level, supervisors’ age, supervisors’ professional title, and the number of master’s students supervised by the supervisor. Graduate students’ gender was categorized as male or female. Grade level was categorized as first-year, second-year, or third-year master’s student. Supervisors’ age was categorized as under 30 years old, 30–39 years old, 40–49 years old, 50–59 years old, and 60 years old or above. Supervisors’ professional title was categorized as professor, associate professor, or lecturer. The number of master’s students supervised was recorded as the actual number reported by the respondent and treated as a continuous covariate. Grade level, supervisor age, professional title, and university were represented using dummy variables in the regression analyses, with first-year students, supervisors aged 40–49 years, professors, and University A used as reference categories, respectively. The 40–49-year-old supervisor group was selected as the reference category because it represented the largest and most central age group in the sample, thereby providing a relatively stable comparison group.

### 2.5 Quantitative data analysis

#### 2.5.1 Reliability, construct validity, and common method variance

The psychometric properties of the three study measures were evaluated using internal consistency analysis, exploratory dimensionality assessment, and confirmatory factor analysis (CFA). Internal consistency was assessed using Cronbach’s α. Exploratory dimensionality was examined using principal component analysis, after evaluating sampling adequacy using the Kaiser–Meyer–Olkin (KMO) statistic and Bartlett’s test of sphericity. Eigenvalues, item-level loadings, cross-loadings, and the cumulative percentage of variance explained were examined. The overall Cronbach’s α was 0.866, while the α coefficients for perceived supervisor guidance, perceived graduate affairs management support, and self-reported research development status were 0.850, 0.841, and 0.843, respectively. The KMO value was 0.885, and Bartlett’s test was significant, χ^2^(78) = 2066.899, *p* < 0.001. Three components with eigenvalues of 5.004, 2.050, and 1.531 were retained, accounting for 66.04% of the total variance. Primary item loadings ranged from 0.745 to 0.829, with no substantial cross-loadings.

CFA was subsequently conducted to evaluate the hypothesized three-factor measurement structure. Model fit was assessed using χ^2^, the comparative fit index (CFI), Tucker–Lewis index (TLI), root mean square error of approximation (RMSEA), and standardized root mean square residual (SRMR). The three-factor model showed satisfactory fit, χ^2^(62) = 60.35, *p* = 0.536, CFI = 1.000, TLI = 1.000, RMSEA = 0.000, and SRMR = 0.028. Standardized factor loadings ranged from approximately 0.687 to 0.779. Composite reliability (CR) values were 0.850, 0.842, and 0.843, and average variance extracted (AVE) values were 0.532, 0.570, and 0.573, respectively. Heterotrait–monotrait ratios (HTMT) ranged from approximately 0.360 to 0.493, providing additional evidence of discriminant validity. Detailed item-level results are reported in Supplementary Tables 2, 3.

Because the three focal constructs were obtained from the same respondents at the same time point using positively worded self-report items, potential common method variance (CMV) was additionally assessed. Harman’s single-factor test showed that the first unrotated factor accounted for 38.49% of the variance. In addition, a one-factor CFA showed substantially poorer fit than the hypothesized three-factor model, χ^2^(65) = 855.03, CFI = 0.608, TLI = 0.530, RMSEA = 0.180, and SRMR = 0.133.

#### 2.5.2 Statistical analysis

Descriptive statistics were used to summarize the distributions of the focal variables, and Pearson correlations were calculated to examine their bivariate associations. Multiple linear regression models were then estimated to examine the associations of perceived supervisor guidance (PSG) and perceived graduate affairs management support (PGAMS) with self-reported research development status (RDS). Adjusted models included student gender, grade level, supervisor age, supervisor professional title, the actual number of master’s students supervised, and university fixed effects. Grade level, supervisor age, supervisor professional title, and university were represented by dummy variables, while the number of master’s students supervised was treated as a continuous covariate. PSG and PGAMS were examined both separately and simultaneously to evaluate their independent associations with RDS.

A cross-sectional statistical indirect association was examined using nonparametric bootstrap resampling with 5,000 bootstrap samples. The analysis estimated the association between PSG and PGAMS, the association between PGAMS and RDS conditional on PSG, the direct association between PSG and RDS conditional on PGAMS, and the resulting indirect association through PGAMS. Unstandardized coefficients (B) and 95% bootstrap confidence intervals were reported. An additional exploratory interaction analysis was conducted to evaluate the alternative possibility that the association between PSG and RDS varied according to PGAMS. PSG and PGAMS were mean-centered before constructing the PSG × PGAMS product term, which was added to the adjusted regression model.

Prior to interpreting the regression models, the analytic data were examined for missing values and model assumptions. Linearity was evaluated using residual diagnostics and the Ramsey RESET test; homoscedasticity was assessed using the Breusch–Pagan and White tests; multicollinearity was assessed using variance inflation factors (VIFs); residual distributions were examined using graphical and normality diagnostics; and influential observations were evaluated using Cook’s distance. Robustness was further examined using HC3 heteroskedasticity-consistent standard errors and sensitivity analyses excluding potentially influential observations. All analyses were conducted using R 4.5.

#### 2.5.3 Latent profile analysis

Latent profile analysis (LPA) was used to identify heterogeneous support configurations based on the nine item-level indicators of perceived supervisor guidance and perceived graduate affairs management support. One- to five-profile solutions were estimated using maximum likelihood estimation with multiple random starting values to reduce the likelihood of convergence on local optima. Specifically, the Akaike information criterion (AIC), Bayesian information criterion (BIC), and sample-size-adjusted BIC (aBIC) were used to compare relative model fit, with lower values indicating better relative fit. Classification quality was evaluated using entropy and average posterior probabilities (AvePP). The adjusted Lo–Mendell–Rubin likelihood ratio test (aLMR-LRT) and the bootstrap likelihood ratio test (BLRT) were used to evaluate whether a model with k profiles provided an improvement over a model with k - 1 profiles. The admissibility of each solution was also examined, including convergence, profile size, separation of indicator patterns, and the absence of boundary or near-singular solutions such as class-specific variances approaching zero. After the final profile solution was selected, profiles were labeled descriptively according to their indicator patterns. Differences in self-reported research development status (RDS) across profiles were evaluated using the Bolck–Croon–Hagenaars (BCH) distal-outcome approach. BCH-adjusted comparisons were conducted for the overall RDS score and its component aspects. Latent profile and distal-outcome analyses were conducted using R 4.5.

### 2.6 Qualitative interviews and analysis

#### 2.6.1 Interview participants

The qualitative component included three groups of participants from the three participating universities: graduate students, supervisors, and graduate affairs administrators. The 18 graduate student interviewees were selected from among participants in the concurrent questionnaire survey, with six students interviewed at each university. The student interview sample covered first-year, second-year, and third-year master’s students. In addition, two supervisors and two graduate affairs administrators were initially recruited from each university. Their disciplinary backgrounds included computer vision, civil engineering, electronic information, mechanical engineering, materials science, environmental engineering, automation control, biomedical engineering, software engineering, chemical engineering, information and communication engineering, transportation engineering, energy and power engineering, artificial intelligence, surveying and remote sensing, food science and engineering, and physical electronics. The initial supervisor interview sample included six participants, covering professors, associate professors, and lecturers, with disciplinary backgrounds in mechanical engineering, computer science, materials science, civil engineering/environmental engineering, and electronic information/automation. One supervisor requested to withdraw after the interview; therefore, five supervisor interviews were retained for analysis. The graduate affairs administrator interview sample included six participants, covering positions such as college-level graduate teaching secretaries, directors of graduate training offices, college-level graduate student affairs administrators, directors of professional degree education centers, and university-level graduate school training administrators. Graduate student interviewees were coded as S01–S18, supervisor interviewees were initially coded as T01–T06, and graduate affairs administrator interviewees were coded as GMA01–GMA06. All interview data were anonymized and de-identified.

#### 2.6.2 Interview procedure

The interviews were conducted in a one-on-one semi-structured format, and each interview lasted approximately 40–45 min. The interview protocol covered supervisor guidance practices and frequency, research resource support, institutional implementation and academic process management, training milestone reminders, life-related services, psychological care, supervisor–student communication, research-related pressure, thesis and graduation risks, and participants’ perceptions of the functions, connections, and role boundaries of supervisor guidance and graduate affairs management support. For graduate student interviews, the focus was placed on specific experiences during research training, including perceived supervisor guidance, perceived graduate affairs management support, research engagement, competence development, research output experiences, research-related pressure, and research–life balance. For supervisor interviews, the focus was placed on research training, academic guidance, resource provision, process feedback, student development, and supervisor–student communication. For graduate affairs administrator interviews, the focus was placed on institutional implementation, training milestone management, academic early warning, student support, psychological care, and coordination surrounding graduate training processes. Before each interview, the researcher explained the purpose of the study, the use of the data, and the principles of anonymity. All participants provided informed consent, including consent for audio recording. The interviews were audio-recorded and subsequently transcribed verbatim in Chinese. Before qualitative analysis, all transcripts were anonymized and de-identified, and identifying information was removed or replaced with participant codes.

#### 2.6.3 Qualitative data analysis

The interview data were analyzed using a deductive–inductive thematic analysis. An initial coding framework was informed by perceived supervisor guidance, perceived graduate affairs management support, research development, and loose coupling theory. The researchers then repeatedly read the Chinese transcripts and conducted open coding to identify additional experiences, events, and explanatory patterns emerging from the data.

Two researchers participated in the coding process. A shared codebook containing code names, definitions, and representative text segments was developed and refined during analysis. When differences in coding interpretation arose, the two researchers discussed the relevant passages and returned to the original transcripts until consensus was reached. Initial codes were subsequently integrated into focused categories and higher-order themes. To address researcher reflexivity, the theory-informed initial framework was treated as a starting point rather than an exhaustive coding structure, and inductively generated codes that captured relevant content beyond the initial framework were retained in the analysis.

Thematic saturation was considered during data collection and analysis. Saturation was considered to have been reached when later interviews no longer generated substantively new codes or themes relevant to the research questions. NVivo 12 was used to organize the transcripts, codes, categories, and themes.

Coding and theme development were conducted entirely in Chinese. Representative quotations selected for presentation in the English-language manuscript were translated into English by the first author and independently checked against the original Chinese transcripts by the other author. The qualitative analysis was used to contextualize and elaborate the quantitative findings by clarifying the differentiated functions, connections, and boundaries of academic and institutional support in graduate research training. Illustrative source excerpts and the progression from initial codes to focused categories and core themes are provided in Supplementary Tables 4, 5.

## 3 Results

### 3.1 Scores for perceived supervisor guidance, perceived graduate affairs management support, and self-reported research development status

The mean scores for perceived supervisor guidance (PSG), perceived graduate affairs management support (PGAMS), and self-reported research development status (RDS) were 3.44 ± 0.82, 3.52 ± 0.84, and 3.31 ± 0.86, respectively. Among the PSG items, guidance competence and timeliness of guidance had the highest mean scores (3.50 ± 1.05 and 3.50 ± 1.06, respectively), followed by supervisory philosophy (3.44 ± 1.03) and guidance style (3.42 ± 1.03), while resource provision had the lowest mean score (3.35 ± 1.04). Among the PGAMS items, psychological care had the highest mean score (3.58 ± 1.01), followed by institutional implementation (3.56 ± 1.02), academic process management (3.49 ± 1.02), and life-related support (3.44 ± 1.01). Among the RDS items, research–life balance (3.34 ± 1.05) and research output (3.33 ± 1.06) had relatively higher mean scores, followed by research engagement (3.32 ± 1.05) and perceived research competence (3.24 ± 1.04). Pearson correlation analysis showed significant positive correlations among the three study constructs (Table 3). PSG was positively correlated with RDS (*r* = 0.305, *p* < 0.001), PGAMS was positively correlated with RDS (*r* = 0.415, *p* < 0.001), and PSG was positively correlated with PGAMS (*r* = 0.407, *p* < 0.001).

**TABLE 3:** Descriptive statistics and correlations among the three study constructs.

Variable	Mean	SD	1	2	3
1. Perceived supervisor guidance (PSG)	3.44	0.82	1		
2. Perceived graduate affairs management support (PGAMS)	3.52	0.84	0.407***	1	
3. Self-reported research development status (RDS)	3.31	0.86	0.305***	0.415***	1

*N* = 378. SD, standard deviation.

***p < 0.001.

### 3.2 Direct and indirect associations among perceived supervisor guidance, perceived graduate affairs management support, and self-reported research development status

#### 3.2.1 Direct associations

Table 4 presents the multiple linear regression results for the associations of PSG and PGAMS with RDS after adjustment for student gender, grade level, supervisor age, supervisor professional title, the actual number of master’s students supervised, and university fixed effects.

**TABLE 4:** Multiple linear regression models predicting self-reported research development status.

Variable	Model 1			Model 2			Model 3			Model 4		
	B	SE	β	B	SE	β	B	SE	β	B	SE	β
Constant	3.397***	0.161	–	2.310***	0.235	–	1.940***	0.221	–	1.613***	0.244	–
Gender: female	0.013	0.102	0.007	0.063	0.098	0.034	−0.012	0.093	−0.007	0.018	0.092	0.010
Grade: second-year	−0.138	0.109	−0.074	−0.159	0.104	−0.086	−0.097	0.099	−0.052	−0.115	0.098	−0.062
Grade: third-year	−0.129	0.108	−0.069	−0.154	0.103	−0.083	−0.078	0.099	−0.042	−0.099	0.098	−0.053
Supervisor age: < 30 years	1.025*	0.424	0.136	0.819*	0.406	0.109	0.630	0.389	0.083	0.587	0.385	0.078
Supervisor age: 30–39 years	0.024	0.115	0.013	−0.013	0.109	−0.007	−0.017	0.104	−0.009	−0.029	0.103	−0.016
Supervisor age: 50–59 years	0.052	0.127	0.025	−0.038	0.122	−0.018	0.065	0.116	0.031	0.017	0.115	0.008
Supervisor age: ≥ 60 years	0.863*	0.336	0.135	0.801*	0.321	0.125	0.875**	0.306	0.137	0.841**	0.303	0.131
Supervisor title: associate professor	0.068	0.116	0.039	0.078	0.111	0.044	0.058	0.106	0.033	0.064	0.105	0.036
Supervisor title: lecturer	−0.198	0.211	−0.058	−0.074	0.202	−0.022	−0.010	0.193	−0.003	0.024	0.192	0.007
Number of master’s students supervised	0.003	0.017	0.010	0.001	0.016	0.003	−0.003	0.015	−0.011	−0.003	0.015	−0.011
University B	−0.142	0.109	−0.078	−0.122	0.104	−0.066	−0.178	0.099	−0.097	−0.162	0.098	−0.088
University C	−0.140	0.116	−0.076	−0.112	0.111	−0.061	−0.228*	0.106	−0.124	−0.200	0.105	−0.108
Perceived supervisor guidance (PSG)	–	–	–	0.321***	0.052	0.306	–	–	–	0.165**	0.055	0.157
Perceived graduate affairs management support (PGAMS)	–	–	–	–	–	–	0.432***	0.049	0.418	0.364***	0.054	0.352
R^2^	0.043			0.132			0.210			0.230		
Adjusted R^2^	0.011			0.102			0.182			0.200		
F	1.363			4.276***			7.463***			7.739***		

*N* = 378. The dependent variable was self-reported research development status (RDS). Reference categories were male for gender, first-year master’s student for grade level, 40–49 years for supervisor age, professor for supervisor professional title, and University A for university affiliation. The number of master’s students supervised was entered as the actual reported number and treated as a continuous covariate. B, unstandardized regression coefficient; SE, standard error; β, standardized regression coefficient.

*p < 0.05;

**p < 0.01;

***p < 0.001.

Model 1 included the control variables and university fixed effects. The model explained 4.3% of the variance in RDS (R^2^ = 0.043, adjusted R^2^ = 0.011) and was not statistically significant overall (*p* = 0.181).

Model 2 added PSG. PSG was positively associated with RDS (*B* = 0.321, SE = 0.052, β = 0.306, *p* < 0.001), and the overall model was statistically significant, *F*(13, 364) = 4.276, *p* < 0.001, R^2^ = 0.132, adjusted R^2^ = 0.102.

Model 3 added PGAMS to the control-variable model. PGAMS was positively associated with RDS (*B* = 0.432, SE = 0.049, β = 0.418, *p* < 0.001), and the overall model was statistically significant, *F*(13, 364) = 7.463, *p* < 0.001, R^2^ = 0.210, adjusted R^2^ = 0.182.

Model 4 included PSG and PGAMS simultaneously. Both PSG (*B* = 0.165, SE = 0.055, β = 0.157, *p* = 0.003) and PGAMS (*B* = 0.364, SE = 0.054, β = 0.352, *p* < 0.001) remained positively associated with RDS, and the overall model was statistically significant, *F*(14, 363) = 7.739, *p* < 0.001, R^2^ = 0.230, adjusted R^2^ = 0.200. These results further supported H1 and H2.

Regression diagnostics did not indicate major violations of the model assumptions. The Ramsey RESET test was not significant (*p* = 0.477), and neither the Breusch–Pagan test (*p* = 0.659) nor the White test (*p* = 0.954) indicated heteroscedasticity. All variance inflation factors were below 1.7, indicating low multicollinearity, and the maximum Cook’s distance was 0.043. The Jarque–Bera test was not significant (*p* = 0.221), whereas the Shapiro–Wilk test indicated a small departure from normality (*p* = 0.026); graphical residual diagnostics did not indicate severe non-normality. Analyses using HC3 heteroskedasticity-consistent standard errors and sensitivity analyses excluding potentially influential observations yielded the same substantive conclusions.

#### 3.2.2 Indirect association through perceived graduate affairs management support

Bootstrap analysis was used to examine the hypothesized cross-sectional indirect association among PSG, PGAMS, and RDS. PSG was positively associated with PGAMS (a path: *B* = 0.429, SE = 0.048, 95% CI [0.334, 0.524], *p* < 0.001), supporting H3. When PSG and PGAMS were entered simultaneously in the RDS model, PGAMS remained positively associated with RDS (b path: *B* = 0.364, SE = 0.054, 95% CI [0.258, 0.469], *p* < 0.001), while PSG retained a positive direct association with RDS (c’ path: *B* = 0.165, SE = 0.055, 95% CI [0.058, 0.272], *p* = 0.003). The total association between PSG and RDS was *B* = 0.321 (SE = 0.052, 95% CI [0.218, 0.424], *p* < 0.001).

The bootstrap indirect association through PGAMS was *B* = 0.156, with a 95% bootstrap CI of [0.103, 0.217]. Because the confidence interval did not include zero, the hypothesized indirect-association pattern was statistically supported, consistent with H4. Given the concurrent measurement of PSG, PGAMS, and RDS, this result is interpreted as a cross-sectional statistical indirect association rather than evidence of temporal or causal mediation (Table 5).

**TABLE 5:** Bootstrap indirect-association analysis.

Association	*B*	SE	95% CI	*p*
PSG → RDS, total association (c)	0.321	0.052	0.218, 0.424	< 0.001
PSG → PGAMS (a)	0.429	0.048	0.334, 0.524	< 0.001
PGAMS → RDS, controlling for PSG (b)	0.364	0.054	0.258, 0.469	< 0.001
PSG → RDS, controlling for PGAMS (c’)	0.165	0.055	0.058, 0.272	0.003
PSG → PGAMS → RDS, indirect association (ab)	0.156	–	0.103, 0.217	–

B, unstandardized coefficient. The confidence interval for the indirect association was obtained from 5,000 bootstrap resamples. Models adjusted for the prespecified covariates and university fixed effects.

Figure 2 summarizes the cross-sectional indirect-association model. PSG was positively associated with PGAMS, and both PSG and PGAMS were positively associated with RDS in the adjusted model. The bootstrap indirect association through PGAMS was statistically significant.

**FIGURE 2:**
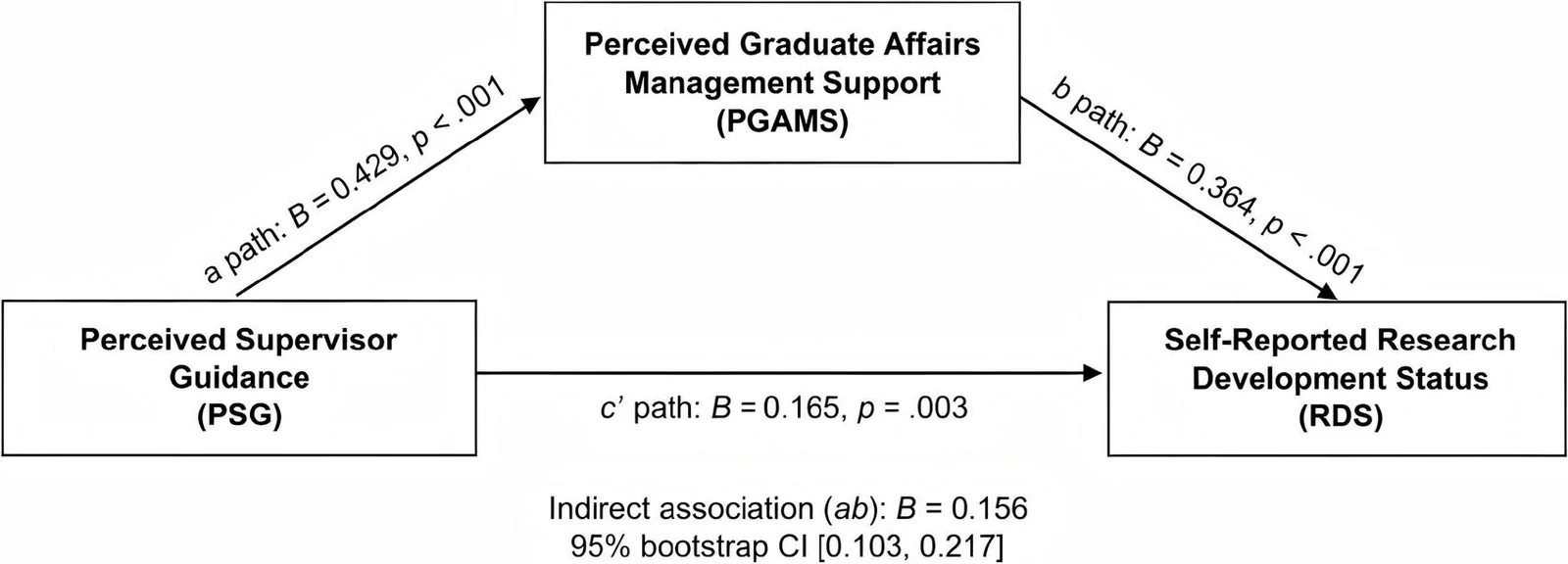
Cross-sectional indirect-association model among PSG, PGAMS, and RDS.

#### 3.2.3 Exploratory interaction analysis

As an additional exploratory analysis, we examined whether PGAMS moderated the association between PSG and RDS. The interaction between mean-centered PSG and PGAMS was not statistically significant (*B* = 0.005, SE = 0.057, 95% CI [-0.107, 0.117], *p* = 0.933) and produced virtually no increase in explained variance (ΔR^2^ < 0.001). Analyses using HC3 heteroskedasticity-consistent standard errors yielded the same conclusion.

### 3.3 Latent support profiles and differences in self-reported research development status

#### 3.3.1 Fit results of the latent profile models

The one- through five-profile solutions were compared using information criteria, classification quality, likelihood-ratio tests, profile size, model admissibility, and substantive interpretability (Table 6). Relative to the three-profile solution (AIC = 8865.2, BIC = 9085.5, aBIC = 8907.9, entropy = 0.849), the four-profile solution showed improved information criteria (AIC = 8672.5, BIC = 8967.6, aBIC = 8729.6) while maintaining satisfactory classification quality (entropy = 0.855). Average posterior probabilities for the four profiles ranged from 0.912 to 0.939, and the smallest profile comprised 13.8% of the sample. In addition, the adjusted Lo–Mendell–Rubin likelihood ratio test comparing the four-profile and three-profile solutions was significant (*p* < 0.001), as was the bootstrap likelihood ratio test (*p* = 0.002).

**TABLE 6:** Fit and classification indices for the latent profile models.

Profiles	AIC	BIC	aBIC	Entropy	Profile sizes	Minimum profile	AvePP range	aLMR-LRT	BLRT	Model status
1	9880.0	9950.8	9893.7	–	378	100.0%	–	–	–	Admissible
2	9103.6	9249.1	9131.8	0.841	199/179	47.4%	0.947–0.961	< 0.001	<0.001	Admissible
3	8865.2	9085.5	8907.9	0.849	93/124/161	24.6%	0.913–0.945	< 0.001	<0.001	Admissible
4	8672.5	8967.6	8729.6	0.855	129/94/103/52	13.8%	0.912–0.939	< 0.001	0.002	Selected
5	7865.2	8235.1	7936.9	0.868	78/41/80/82/97	10.8%	0.870–0.982	NI	NI	Near-singular/inadmissible

AIC, Akaike information criterion; BIC, Bayesian information criterion; aBIC, sample-size-adjusted BIC; AvePP, average posterior probability; aLMR-LRT, adjusted Lo–Mendell–Rubin likelihood ratio test; BLRT, bootstrap likelihood ratio test; NI, not interpreted because the solution was inadmissible. In the five-profile solution, at least one class-specific indicator variance converged to approximately 1 × 10^−6^, indicating variance collapse. The four-profile solution was therefore retained.

Although the five-profile solution produced substantially lower information criteria and a slightly higher entropy value, inspection of the parameter estimates indicated a near-singular solution, with at least one class-specific indicator variance converging to the numerical lower bound (approximately 1 × 10^−6^). This variance collapse indicated that the apparent improvement in fit was associated with an inadmissible boundary solution and was therefore not interpreted as evidence for a substantively valid fifth profile. Considering statistical fit, classification quality, model admissibility, profile size, and substantive interpretability together, the four-profile solution was retained as the final model (Supplementary Figure 1 and Supplementary Table 6).

#### 3.3.2 Naming and characteristics of the support profiles

Based on the estimated patterns of perceived supervisor guidance (PSG) and perceived graduate affairs management support (PGAMS), the four profiles were labeled fully supported, management-support-dominant, supervisor-support-dominant, and insufficiently supported (Figure 3). The fully supported profile comprised 129 students (34.1%) and was characterized by high levels of both PSG and PGAMS. The estimated overall means were approximately 4.25 for PSG and 4.19 for PGAMS, indicating consistently favorable perceptions across both support domains. The management-support-dominant profile comprised 94 students (24.9%). Students in this profile reported a comparatively lower level of PSG (M ≈ 2.89) but a substantially higher level of PGAMS (M ≈ 3.97). The supervisor-support-dominant profile comprised 103 students (27.2%) and showed the opposite configuration: PSG was relatively high (M ≈ 3.55), whereas PGAMS was comparatively lower (M ≈ 2.84). This profile demonstrates that stronger perceived supervisor guidance can coexist with less favorable perceptions of institutional and process-based support. The insufficiently supported profile comprised 52 students (13.8%) and showed comparatively low levels of both PSG (M ≈ 2.26) and PGAMS (M ≈ 2.50). Taken together, the four-profile solution revealed substantial heterogeneity in how master’s students experienced the two support domains.

**FIGURE 3:**
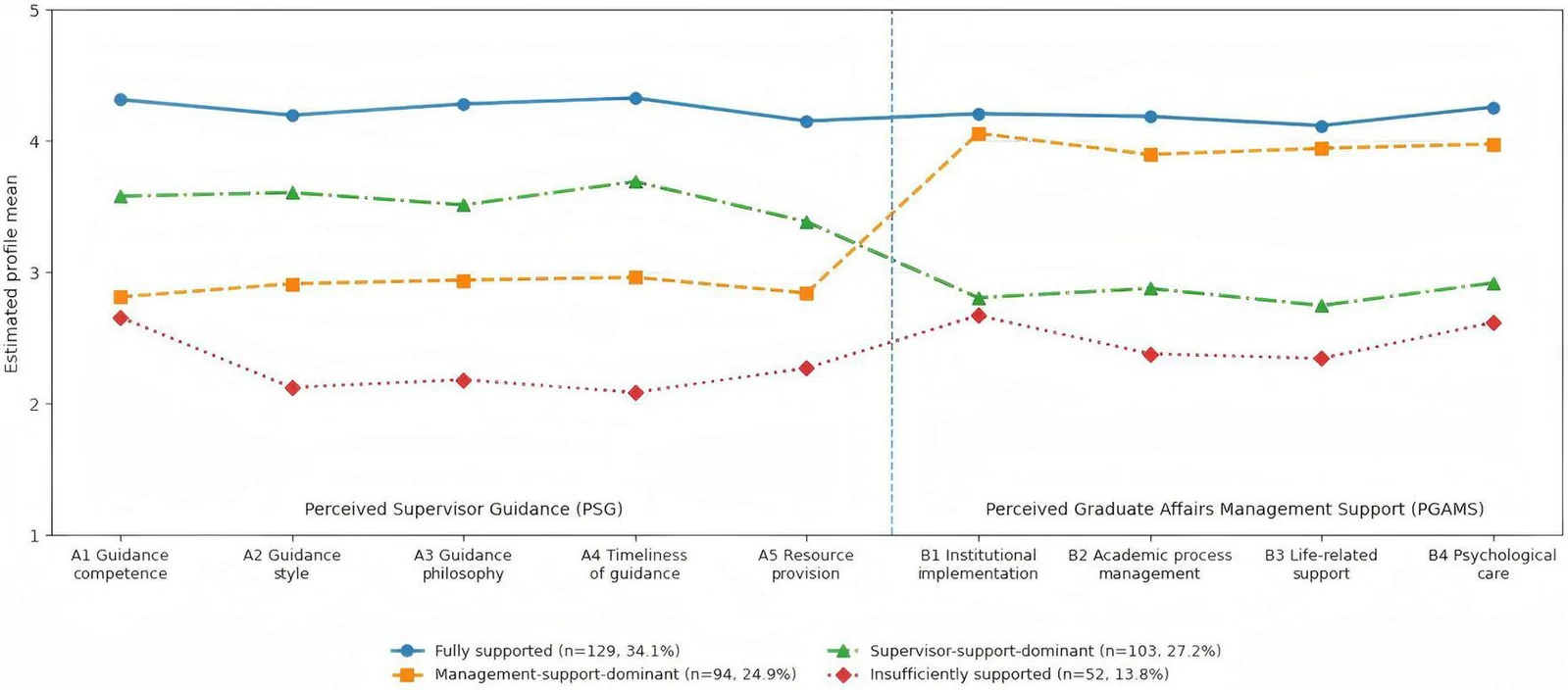
Profile means across the four latent support profiles. A1–A5 represent the five perceived supervisor guidance (PSG) indicators, and B1–B4 represent the four perceived graduate affairs management support (PGAMS) indicators. The *y*-axis represents the estimated profile-specific mean on the 1–5 response scale. Lines connect the estimated means across indicators for visualization, and the vertical dashed line separates the PSG and PGAMS indicator domains.

#### 3.3.3 Differences in research development status across support types

To account for uncertainty in latent-profile classification, differences in self-reported research development status (RDS) were examined using the Bolck–Croon–Hagenaars (BCH) distal-outcome approach. The BCH-adjusted results showed significant overall differences across the four profiles in total RDS and in each of its four component aspects (all omnibus *p* < 0.001; Table 7).

**TABLE 7:** BCH-adjusted research development outcomes across the four latent support profiles.

RDS indicator	Fully supported	Management-support-dominant	Supervisor-support-dominant	Insufficiently supported	Overall *p*
Research engagement	3.78	3.37	3.02	2.70	< 0.001
Perceived research competence	3.66	3.36	2.87	2.75	< 0.001
Research output	3.66	3.46	3.07	2.84	< 0.001
Research–life balance	3.87	3.33	2.94	2.89	< 0.001
Total RDS score	3.74	3.38	2.98	2.80	< 0.001

Values are BCH-adjusted distal-outcome means and account for uncertainty in latent-profile classification. RDS = self-reported research development status. Overall *p*-values refer to omnibus BCH comparisons across the four profiles.

For total RDS, the adjusted mean was highest in the fully supported profile (M ≈ 3.74), followed by the management-support-dominant profile (M ≈ 3.38) and the supervisor-support-dominant profile (M ≈ 2.98), and was lowest in the insufficiently supported profile (M ≈ 2.80). The same general ordering was observed for research engagement, perceived research competence, research output, and research–life balance. These results indicate that heterogeneous configurations of perceived academic and institutional support were associated with differences in students’ self-reported research-development experiences.

### 3.4 Qualitative findings

#### 3.4.1 Open coding: from interview statements to initial concepts

The open coding results showed that the initial concepts derived from the interview data were concentrated in three broad domains. The first domain concerned supervisor guidance practices, including clarification of research directions, methodological training, experimental guidance, thesis and manuscript revision, submission feedback, research group meeting routines, and the provision of research resources. The second domain concerned graduate affairs management support practices, including interpretation of training plans, reminders about training milestones, arrangements for proposal defense and mid-term assessment, thesis review and defense procedures, graduation review, scholarship and financial aid support, psychological care, and risk identification. The third domain concerned students’ research-development experiences, including variation in research engagement, delayed research progress, competence development, research-output pressure, experimental failure, graduation anxiety, communication avoidance, and difficulties in seeking help (Supplementary Table 4).

Taken together, the initial codes distinguished academic and research-training experiences primarily associated with supervisor guidance from procedural, informational, and student-support experiences associated with graduate affairs management support, while also capturing students’ experiences of research progress, competence development, pressure, and help seeking.

#### 3.4.2 Focused coding: from initial concepts to category integration

Based on the open coding results, the initial concepts were further compared and integrated into six focused categories: supervisor academic guidance, research process training, training process management, student support services, support gaps, and research pressure and development experiences.

“Supervisor academic guidance” included topic-selection support, clarification of research directions, methodological training, experimental guidance, thesis revision, and resource provision. “Research process training” included research group meeting routines, one-on-one discussions, project-task breakdown, stage-based feedback, and peer collaboration. “Training process management” included interpretation of training plans, proposal-defense reminders, mid-term assessment, thesis review and defense, and graduation review. “Student support services” included scholarship and financial aid support, life-related support, psychological care, academic early warning, and risk identification. “Support gaps” included delayed supervisor feedback, unclear requirements or procedures, unclear help-seeking channels, and communication avoidance. “Research pressure and development experiences” included changes in research engagement, competence development, research-output pressure, graduation anxiety, and research–life imbalance (Supplementary Table 5).

#### 3.4.3 Theme integration: from focused categories to core themes

Based on the focused coding results, the six categories were integrated into four core themes: (1) academic entry and research training through supervisor guidance; (2) procedural assurance and risk identification through graduate affairs management support; (3) uncertainty and pressure under support gaps; and (4) institutional support functions and boundaries.

The four themes reflected differentiated but interconnected aspects of students’ support environments. Supervisor guidance was described primarily in relation to research content, methodological training, feedback, and academic judgment, whereas graduate affairs management support was described primarily in relation to training procedures, institutional milestones, student services, and risk identification. Experiences of support gaps were associated in participants’ accounts with uncertainty about academic progress, institutional procedures, help seeking, and graduation-related pressure (Table 8).

**TABLE 8:** Theme generation results: focused categories and core themes.

Core theme	Source categories	Theme content
Academic entry and research training through supervisor guidance	Supervisor academic guidance; research process training	Supervisor guidance was described as supporting research entry, methodological learning, feedback, academic judgment, and competence development.
Procedural assurance and risk identification through graduate affairs management support	Training process management; student support services	Graduate affairs management support was described as providing procedural clarification, milestone reminders, student services, and risk identification throughout graduate training.
Uncertainty and pressure under support gaps	Support gaps; research pressure and development experiences	Participants described unclear academic direction, procedures, and help-seeking channels alongside delayed progress, research-related pressure, and graduation anxiety.
Institutional support functions and boundaries	Training process management; student support services; support gaps	Institutional support could clarify procedures, connect students with services, and identify risks, but responsibility for substantive academic judgment and research guidance remained with supervisors.

#### 3.4.4 Theme 1: academic entry and research training through supervisor guidance

The first theme consisted of two focused categories: supervisor academic guidance and research process training. The interview data showed that, in students’ narratives, supervisor guidance was mainly reflected in the clarification of research directions, methodological training, experimental guidance, thesis revision, and the arrangement of research routines. Supervisor interviews also indicated that research group meetings, one-on-one discussions, project tasks, and thesis revision were recurring forms of everyday guidance.

In supervisor interviews, research group meetings were described as an important way to maintain students’ research pace. One supervisor stated, “In research group meetings, every student has to report their progress… At minimum, they need to explain what they did this week, what problems they encountered, and what they plan to do next week” (T01). Another supervisor distinguished between literature presentations and project progress reports, emphasizing that literature presentations were not merely about “translating papers”; rather, students needed to explain the research problem, methodological innovation, and implications for their own projects (T02).

Similar descriptions also appeared in graduate student interviews. When describing the entry stage in the first year of the master’s program, S01 noted that the supervisor helped divide the current tasks into several parts, including building a course foundation, reading classical models, reproducing a baseline, and comparing candidate research topics. The student stated, “In this way, I was not as confused at the beginning” (S01). In contrast, S02 mentioned that during a stage when supervisor guidance was limited and assistance mainly came from senior peers, the student knew that effort was needed but “did not know whether the path of my effort was correct” (S02). These materials indicate that supervisor guidance was understood in the interviews not only as specific technical assistance, but also as a process through which students clarified research pathways and developed research judgment.

Supervisor guidance was also reflected in experimental design, data interpretation, and the analysis of failed attempts. S04 reported that the supervisor often emphasized that “experimental failure is not frightening; what is frightening is not knowing why it failed and not recording the failure process” (S04). T03 also noted that, in materials-related experiments, students should not only report successful results; failed experiments also needed to be explained and discussed. These interview materials suggest that, in participants’ experiences, supervisor guidance mainly centered on research entry, process training, and competence formation.

#### 3.4.5 Theme 2: procedural assurance and risk identification through graduate affairs management support

The second theme consisted of two focused categories: training process management and student support services. The interview data showed that graduate affairs management support was mainly concentrated on training process management, explanation of institutional information, reminders about key milestones, graduation review, academic early warning, and psychological risk identification.

In interviews with graduate affairs administrators, training process management was described as work that ran through the entire graduate training process. GMA01 stated, “After graduate students enroll, training plan formulation, course selection, credit review, implementation of training plans, proposal defense arrangements, mid-term assessment, academic activity records, thesis pre-defense, thesis review, defense qualification review, preparation of degree application materials, and archiving of graduation materials are basically all related to my work” (GMA01). This statement indicates that graduate affairs management support was first reflected in the continuous management of training procedures and degree milestones.

In graduate student interviews, students described the role of graduate affairs management support more from the perspective of their own experiences. S02 mentioned that the supervisor was relatively busy and could not remind the student of every milestone, whereas notifications from graduate affairs administrators helped the student know which tasks had to be completed on time (S02). When discussing the proposal defense stage, S03 stated, “After the administrator explained the process clearly, many unknowns became known. The tasks still had to be done, but psychologically I felt less panicked” (S03). These accounts show that students experienced procedural clarification as reducing uncertainty surrounding key training milestones.

Across administrator interviews, risk identification was described as an additional function of graduate affairs management support, particularly when academic deadlines, graduation requirements, employment, and psychological pressure overlapped. GMA01 noted that final-year students faced substantial pressure when thesis review, blind review, defense, and employment overlapped, and some students might experience anxiety, insomnia, avoidance of material submission, or reluctance to check review comments. GMA04 also mentioned that milestones such as proposal defense, mid-term assessment, thesis review, defense, graduation, and employment could affect students’ psychological state and research pace. Therefore, colleges needed to provide reminders in advance and offer categorized support for students facing graduation-related risks. These materials indicate that graduate affairs management support involved not only institutional procedures, but also the identification of and response to student developmental risks.

#### 3.4.6 Theme 3: uncertainty and pressure under support gaps

The third theme consisted of two focused categories: support gaps and research pressure and development experiences. The interview data showed that when supervisor guidance, graduate affairs management support, or the connection between the two was insufficient, students were more likely to report uncertainty regarding research direction, training procedures, and graduation requirements. In some students’ narratives, such uncertainty was reflected in delayed research progress, increased anxiety, and communication avoidance.

In graduate student interviews, first-year accounts highlighted unclear research directions and uncertain pathways during the research entry stage. S02 stated that although the student understood the practical significance of structural safety and bridge monitoring, the student “did not know how graduate research should actually begin” and “did not know what I was supposed to gain after reading a paper” (S02). During the proposal defense stage, S03 reported concerns about whether the research topic was sufficiently focused, whether the innovation was adequate, and whether the technical route was feasible. The student stated, “I used to know only that I had to prepare for the proposal defense, but I did not know what I should do every day” (S03). These statements suggest that, during the research entry and proposal defense stages, students’ uncertainty centered on the formation of research questions and the breakdown of stage-based tasks.

Among second- and third-year interviewees, accounts also highlighted pressure related to experimental progress, research output, and graduation milestones. When describing unsatisfactory fatigue life test results, S04 mentioned that because the testing cycle was long, equipment reservations were difficult, and the experimental results could not be explained, the student felt “extremely anxious” (S04). Some third-year students also mentioned that when manuscript submission, thesis review results, employment arrangements, and graduation timing overlapped, their research engagement and life rhythm were affected. Interviews with graduate affairs administrators similarly indicated that final-year students could experience psychological pressure and avoidance behaviors when thesis review, blind review, defense, and employment overlapped.

#### 3.4.7 Theme 4: institutional support functions and boundaries

The fourth theme concerned the functions and boundaries of institutional support. Across student, supervisor, and graduate affairs administrator interviews, graduate affairs management support was described primarily in terms of procedural clarification, information provision, resource coordination, student services, and risk identification. At the same time, participants consistently distinguished these functions from supervisors’ responsibility for substantive academic guidance, research judgment, and thesis quality.

Graduate student accounts illustrated this distinction. S03 explained that uncertainty during the proposal-defense stage concerned issues such as whether the topic could be adjusted, what questions the committee might ask, and whether incomplete materials would affect subsequent training. After the graduate affairs administrator explained the procedure, “many unknowns became known” (S03). S04 similarly noted that although graduate affairs administrators could not solve technical research problems, they could clarify the purpose and requirements of assessment and explain relevant procedural expectations (S04). These accounts indicate that institutional support was experienced as useful for procedural clarity and access to appropriate support, rather than as a substitute for academic guidance.

Supervisor interviews made the distinction even more explicit. T01 stated, “Supervisors are the primary responsible persons for academic training. They are mainly responsible for research direction, research training, thesis quality, and academic norms… Administrators are providers of institutional support and developmental support, responsible for process management, resource coordination, risk identification, and service assurance” (T01). T02 noted that supervisors could observe students’ research performance but might not always be aware of students’ life-related circumstances. When students were out of contact for extended periods, repeatedly absent from research-group meetings, or at risk of delayed graduation, communication with college-level graduate affairs administrators became important (T02). T03 similarly stated that when difficulties involved psychological pressure or life-related problems, students might be referred to counselors, graduate affairs administrators, or psychological support services because “supervisors can provide care, but not all problems can be solved by supervisors” (T03).

Graduate affairs administrators described similar role boundaries. GMA03 reported that graduate training offices communicated with supervisors, provided reminders regarding training milestones, and clarified graduate-training regulations. GMA04 emphasized the relevance of student support, psychological safety, and the organizational environment to students’ ability to remain engaged in graduate training. Taken together, these accounts characterize supervisor guidance and graduate affairs management support as functionally differentiated but connected forms of support: institutional support can clarify procedures, coordinate services, and identify risks, whereas substantive academic judgment and research guidance remain primarily within the supervisor’s role.

## 4 Discussion

### 4.1 Main findings of this study

Drawing on loose coupling theory, this study examined master’s research development from a support-ecology perspective that incorporates both perceived supervisor guidance (PSG) and perceived graduate affairs management support (PGAMS). Taken together, the quantitative and qualitative findings indicate that these two forms of support are functionally differentiated yet interconnected contextual resources within graduate research training. Both PSG and PGAMS were positively associated with self-reported research development status (RDS) when considered simultaneously, suggesting that students’ research-development experiences are related not only to academic guidance from supervisors but also to the broader institutional and process-based support surrounding graduate training. This interpretation is consistent with previous research showing that perceived supervisory and research-community support is closely related to graduate students’ research engagement, innovation, and research productivity (Shang et al., 2024).

The findings also clarify the relationship between the two support domains. The significant cross-sectional indirect association through PGAMS indicates a statistical connection among PSG, PGAMS, and RDS. Moreover, the nonsignificant PSG × PGAMS interaction provides no evidence that PGAMS functions as a conventional multiplicative moderator of the association between PSG and RDS. The four-profile solution identified fully supported, management-support-dominant, supervisor-support-dominant, and insufficiently supported configurations. This pattern extends prior person-centered work showing that postgraduate students may experience qualitatively different combinations of supervisory and broader research-environment support (Cornér et al., 2023).

The qualitative findings further contextualized these quantitative patterns. Participants described supervisor guidance primarily in relation to research direction, methodological training, feedback, and substantive academic judgment, whereas graduate affairs management support was described mainly in relation to procedures, milestone information, student services, coordination, and risk identification. This functional differentiation extends the conventional supervisor–student dyadic perspective toward a multi-actor support ecology for graduate research learning and development. From an educational-psychology perspective, this matters because perceived support constitutes part of the learning environment in which students develop research competence, sustain research engagement, and respond to research-related demands. Prior studies have similarly linked supervisory support with research self-efficacy, innovative research behavior, and postgraduate research productivity, suggesting that support environments may have psychological and developmental significance beyond their immediate organizational functions (Al Makhamreh and Kutsyuruba, 2021).

### 4.2 Perceived support as a context for graduate research learning and development

Graduate research development can be understood not only in terms of observable outputs but also as a learning and developmental process involving sustained research engagement, the development of research competence, and the management of research-related demands alongside personal life. In this sense, the positive associations of PSG and PGAMS with RDS are consistent with an educational-psychology view in which students’ perceptions of their support environment constitute part of the context in which research learning takes place. Previous studies have similarly shown that perceived supervisor support is related to research innovation, research engagement, and postgraduate research productivity, while work–life balance constitutes an important component of graduate students’ broader developmental experience (Gooding et al., 2023).

Supervisor guidance is particularly relevant to this learning environment because it provides students with academic direction, methodological feedback, opportunities for independent research, and access to disciplinary resources. The significance of supervisory support may extend beyond the immediate provision of research assistance. For example, perceived supervisor support has been linked to graduate students’ creative self-efficacy and research innovation, while the quality of the supervisor–student relationship has been associated with academic self-efficacy and learning adaptation (Madan, 2021). These psychological processes were not directly measured in the present study and therefore should not be interpreted as tested mechanisms here; rather, they illustrate plausible learning-related processes through which perceived academic support may matter for graduate research development.

PGAMS represents a different part of the students’ support environment. Graduate education is highly milestone-oriented, and students must navigate requirements such as proposal approval, mid-term assessment, thesis review, defense, and degree completion. Prior work has shown that transparent milestone information and systematic progress monitoring can help graduate programs identify delayed progress and clarify requirements for students and advisors (Young et al., 2024). Research on postgraduate students’ perceived service quality likewise indicates that institutional and service experiences constitute a distinct dimension of the postgraduate educational environment (Haley, 2023). Accordingly, the present findings are better interpreted as showing that academic guidance and institutional/process-based support constitute differentiated contextual resources within the same research-learning environment, rather than as evidence that one form of support is inherently more important than the other.

### 4.3 Differentiated functions of supervisor guidance and graduate affairs management support in research development

The positive association between perceived supervisor guidance (PSG) and self-reported research development status (RDS) is consistent with the central role of supervision in master’s research training. Supervisors contribute directly to students’ engagement with research content through topic clarification, methodological guidance, feedback on research problems, thesis development, and access to disciplinary resources. A recent review of supervision similarly identified supervisor actions, feedback, and the quality of the student–supervisor relationship as important elements of students’ learning and research outcomes (Huet and Casanova, 2022). In this sense, PSG primarily reflects the academic and research-training conditions under which students learn to formulate questions, develop methods, evaluate evidence, and progressively undertake research with greater independence.

Perceived graduate affairs management support (PGAMS) represents a different form of support. It is oriented toward the institutional and process conditions surrounding research training, including clarification of regulations, milestone management, access to student services, coordination, and risk identification. Graduate programs require students to navigate a sequence of formal milestones, and transparent monitoring of these milestones can make progress requirements more visible to both students and programs (Suri-Ogilvie, 2024). More broadly, previous work has argued that universities need support structures beyond the supervisor alone, including transparent program expectations, researcher-development resources, and institutional services that supervisors and students can draw on when needs extend beyond substantive research guidance (Gupta et al., 2022). PGAMS can therefore be understood as contributing primarily to the procedural clarity, organizational accessibility, and continuity of support surrounding students’ research activities.

The fact that PSG and PGAMS both remained positively associated with RDS when entered simultaneously is therefore better interpreted as evidence of functional differentiation. Supervisor guidance addresses substantive questions of research direction, methods, feedback, and academic standards, whereas graduate affairs management support addresses training procedures, institutional requirements, service access, and coordination. This distinction is also compatible with loose coupling theory: different actors within graduate training may retain relatively autonomous responsibilities while remaining connected through shared training processes. Accordingly, the present findings indicate that students’ research development is situated within a support structure in which academic and institutional resources can be distinct yet mutually relevant. This interpretation is consistent with prior work emphasizing that effective graduate education depends both on high-quality supervision and on wider university-level support structures rather than on a single actor assuming responsibility for all aspects of students’ development (McCain et al., 2024).

### 4.4 Interconnections between supervisor guidance and graduate affairs management support

The significant cross-sectional indirect association through perceived graduate affairs management support (PGAMS) indicates that perceived supervisor guidance (PSG), PGAMS, and self-reported research development status (RDS) are statistically connected within the same graduate-training environment. One plausible interpretation is that PSG and PGAMS partly reflect students’ experiences of connected but distinct support arrangements within the same training system. Supervisors and graduate affairs units may interact around training milestones, procedural requirements, delayed progress, or student-support needs, while retaining different responsibilities. Previous work has similarly emphasized that supervision does not operate independently of the wider institutional environment: universities contribute program structures, support services, researcher-development resources, and procedural expectations that can shape students’ overall supervisory experience (Crook et al., 2021). This interpretation is also compatible with loose coupling theory, which allows organizational actors to remain relatively autonomous while still becoming connected around shared tasks and institutional processes (Bloch, 2021).

The additional interaction analysis further helps delimit this interpretation. The PSG × PGAMS interaction was not statistically significant, indicating that the association between PSG and RDS did not systematically vary across levels of PGAMS in the conventional multiplicative sense. Instead, the quantitative and qualitative evidence is more consistent with a structure in which academic and institutional support are related but functionally differentiated: institutional support may clarify procedures, coordinate services, and identify risks, whereas substantive research direction, methodological judgment, and academic standards remain primarily within the supervisor’s role.

### 4.5 Heterogeneous support configurations and differences in research development

The four-profile solution highlights substantial heterogeneity in how master’s students experience academic and institutional support. The fully supported profile combined relatively high perceived supervisor guidance (PSG) and perceived graduate affairs management support (PGAMS), whereas the insufficiently supported profile combined comparatively low levels of both. The BCH-adjusted results showed that students in the fully supported profile reported the most favorable overall research-development status, while those in the insufficiently supported profile reported the least favorable outcomes. This general pattern is consistent with previous person-centered research showing that doctoral students who experience higher levels of support from supervisors and the wider research environment tend to report stronger research engagement and more favorable study experiences than those in lower-support profiles (Wang et al., 2023).

More importantly, the present study identified two asymmetric support configurations: a management-support-dominant profile and a supervisor-support-dominant profile. These profiles suggest that academic and institutional support do not necessarily rise or fall together. This finding extends earlier profile-based studies in which supervisor and research-community support tended to cluster mainly into generally high, moderate, or low levels (Pyhältö et al., 2023). In the present data, the management-support-dominant profile showed a higher BCH-adjusted RDS mean than the supervisor-support-dominant profile. The interpretation is that students may encounter distinct combinations of academic and institutional resources, and that these combinations are associated with different research-development experiences.

The four-profile structure also helps clarify the practical significance of low-support environments. Students in the insufficiently supported profile reported the lowest overall RDS and comparatively low scores across research engagement, perceived research competence, research output, and research–life balance. Similar profile-based studies have found that students experiencing reduced supervisory and research-community support report lower engagement and satisfaction and higher levels of stress, burnout, or dropout intention than peers with more adequate support (Jackman et al., 2023). These findings suggest that the value of the person-centered analysis lies not in assigning students to fixed labels, but in identifying patterns of support imbalance or insufficiency that may warrant closer attention in graduate training.

### 4.6 Qualitative contextualization of differentiated support functions

The qualitative findings provide contextual detail on how academic and institutional support were experienced in everyday graduate research training. With respect to supervisor guidance, participants referred to clarification of research directions, methodological training, experimental guidance, manuscript feedback, research-group interaction, and access to research resources. These accounts are consistent with previous work showing that mismatches in supervisor–student expectations, as well as clarity in guidance, can shape research-training experiences and research-skill development (Cardilini et al., 2022). In the present interviews, supervisor guidance was therefore described primarily as support for entering research practice, developing research competence, and making substantive academic judgments.

Graduate affairs management support was described in a different way. Participants emphasized training-plan interpretation, proposal and mid-term procedures, thesis review and defense requirements, graduation review, financial and life-related support, psychological care, and risk identification. These accounts suggest that uncertainty in graduate training may arise not only from research tasks but also from unclear procedures, milestones, service access, and overlapping academic or personal pressures (Aldhi et al., 2026). Prior work likewise emphasizes that universities have responsibilities beyond the supervisor alone and that institutional structures and support services form part of the broader postgraduate training environment (Alshayhan et al., 2023). Research on postgraduate researchers has also shown that academic pressures and available social support are relevant to students’ well-being and coping experiences during research training (Acharya and Rajendran, 2023). In this study, PGAMS was therefore better understood as providing procedural clarity, service access, coordination, and risk identification.

Taken together, the qualitative findings help interpret the quantitative results by showing why PSG and PGAMS may be statistically related while retaining different functions. Supervisors, graduate affairs administrators, and students are connected through shared training processes such as proposal development, milestone review, thesis assessment, and graduation requirements, but the interviews consistently indicated that responsibility for research direction, methodological judgment, and academic standards remained primarily with supervisors. This pattern is compatible with the idea of a loosely coupled support structure in which different actors retain relatively autonomous responsibilities while interacting around common training tasks.

### 4.7 Practical implications

The findings of this study have several practical implications for the governance of graduate training in science and engineering universities.

First, universities should develop a multi-actor support structure rather than relying on either supervisors or administrative units alone. The present findings suggest that academic guidance and institutional/process-based support are distinct but mutually relevant components of students’ research-training environment. Universities can therefore clarify the responsibilities of supervisors, graduate affairs administrators, research teams, and relevant student-support services, while establishing communication procedures for issues that cross these functional areas (Trihantoyo et al., 2023). Such arrangements are particularly important around key training stages, including research entry, proposal development, mid-term assessment, thesis review, and degree completion. Previous work similarly argues that effective postgraduate research training requires both high-quality supervision and broader institutional support structures rather than placing responsibility for all aspects of student development on individual supervisors (Akala and Akala, 2023).

Second, universities should continue to strengthen the quality of supervisor guidance as an academic function. Supervisor development can place greater emphasis on research-direction clarification, methodological feedback, progress monitoring, thesis and manuscript feedback, academic norms, and communication about expectations. Institutions can also provide continuing professional development opportunities (Wardhani et al., 2026) and recognize high-quality supervision as an important component of academic work, rather than evaluating supervision primarily through students’ final research outputs. Existing supervisory-practice frameworks and research on supervisor development similarly emphasize supporting candidates’ research projects, providing appropriate feedback, monitoring progress, and continuously enhancing supervisory practice (Jara, 2021).

Third, graduate affairs management support should extend beyond routine procedural notification toward transparent process support, service coordination, and appropriate referral. This does not mean transferring academic responsibilities from supervisors to administrators. Rather, graduate affairs units can improve the clarity of training requirements and timelines, provide timely milestone information, identify students who encounter prolonged procedural or progression difficulties, and connect students with appropriate academic, financial, career, or psychological support services when needs exceed the responsibilities of a single actor. This broader institutional role is consistent with recommendations that supervisors should be able to draw on university-level support systems rather than being expected to address every dimension of postgraduate students’ development and well-being themselves (Löfström et al., 2023).

Fourth, universities may use the identified support configurations to inform differentiated support strategies. For students displaying a pattern similar to the fully supported profile, universities can focus on maintaining existing support while expanding opportunities for advanced research participation, academic exchange, and scholarly development. For students whose experiences resemble the management-support-dominant profile, priority can be given to strengthening access to substantive academic guidance, including research-direction clarification, methodological feedback, and thesis support. Conversely, students resembling the supervisor-support-dominant profile may benefit from clearer information about institutional procedures, milestone requirements, student services, and available support channels. For students showing a pattern of insufficient support across both domains, earlier coordinated attention from supervisors, graduate affairs administrators, and relevant university support services may be appropriate. Person-centered studies similarly indicate that students experiencing lower or imbalanced levels of supervisory and research-community support can differ meaningfully in engagement, stress, satisfaction, and other study experiences (Cano et al., 2024).

### 4.8 Limitations and future research directions

This study has three main limitations. First, the quantitative component used a cross-sectional, single-source self-report design. PSG, PGAMS, and RDS were measured concurrently; therefore, the observed direct and indirect associations cannot establish temporal ordering or causal mediation. RDS reflects students’ self-evaluations rather than independently verified performance. Although the measurement checks did not indicate a dominant common factor and the three brief, context-adapted measures showed satisfactory reliability and construct validity in this sample, these findings do not eliminate method bias. Future studies could use longitudinal, multi-source designs and combine student reports with supervisor assessments, administrative records, objective research outputs, and further measurement validation.

Second, the sample was drawn from three science and engineering universities in Zhejiang Province, which limits generalizability across regions, institutional types, and disciplines. University fixed effects were included in the regression models, but identifiable supervisor IDs were not retained; consequently, possible clustering of students within supervisors could not be modeled directly. The small number of participating universities also limited the feasibility of institution-level multilevel estimation. Future studies should recruit more diverse institutions and, where ethically permissible, retain anonymized linkage identifiers to support multilevel analyses at the student, supervisor, and institutional levels.

Third, the person-centered and mixed-methods components involve residual uncertainty. The BCH approach reduced bias from classification uncertainty in distal-outcome comparisons, but such uncertainty cannot be eliminated completely. In addition, although student interviewees were drawn from survey participants and data collection occurred concurrently, de-identification prevented reliable case-level linkage between interviews and subsequent profile membership. Future mixed-methods studies could preserve consented linkage identifiers and use prospective profile-based sampling and joint displays to strengthen integration across data sources.

## 5 Conclusion

Drawing on loose coupling theory, this study examined master’s students’ research development through a support-ecology perspective incorporating perceived supervisor guidance (PSG) and perceived graduate affairs management support (PGAMS). Both forms of support were independently and positively associated with self-reported research development status (RDS), while the significant indirect association through PGAMS indicated statistical interconnectedness between academic and institutional support. The latent profile analysis further identified four heterogeneous configurations: fully supported, management-support-dominant, supervisor-support-dominant, and insufficiently supported. BCH-adjusted comparisons showed systematic differences in RDS across these configurations, with the fully supported profile showing the most favorable overall research-development pattern and the insufficiently supported profile the least favorable. The qualitative findings complemented these results by showing that supervisor guidance was primarily associated with substantive academic and research-training functions, whereas graduate affairs management support was mainly associated with procedural clarification, service coordination, and risk identification. Overall, the study extends the conventional supervisor–student dyadic perspective by conceptualizing graduate research development as embedded in a multi-actor support ecology. Strengthening graduate training therefore requires not only high-quality academic supervision, but also transparent institutional processes, accessible support services, and effective coordination among the actors involved in students’ research development.

## Data Availability

The raw data supporting the conclusions of this article will be made available by the authors, without undue reservation.
