# Supplementary material for: Support ecology in master’s research development: perceived supervisor guidance and graduate affairs management support
Source: Front Psychol. 2026 Oct 20;17:1927035. doi: 10.3389/fpsyg.2026.1927035 (PMC13727615; doi:10.3389/fpsyg.2026.1927035)
Supplement: Supplementary file 1 [file Supplementary_file_1.pdf]

Supplementary Material

Supplementary Tables S1–S6 and Supplementary Figure S1

Supplementary Table S1. Original Chinese Questionnaire Items, English Translations, Construct Assignment, and Response Scales

Item	Construct	Aspect	Original Chinese wording	English translation	Response scale
A1	PSG	Guidance competence	您对导师的学术指导能力，即帮助您明确研究方向与思路的满意程度。	Your satisfaction with your supervisor’s academic guidance competence, that is, their ability to help you clarify research directions and ideas.	1 = very dissatisfied; 2 = dissatisfied; 3 = neutral; 4 = satisfied; 5 = very satisfied
A2	PSG	Guidance style	您对导师指导方式的满意程度，如是否灵活、因材施教、能有效促进学习与研究。	Your satisfaction with your supervisor’s guidance style, including whether it is flexible, individualized, and effective in facilitating learning and research.	1 = very dissatisfied; 2 = dissatisfied; 3 = neutral; 4 = satisfied; 5 = very satisfied
A3	PSG	Supervisory philosophy	您对导师指导理念的满意程度，如是否注重培养独立研究能力与学术规范。	Your satisfaction with your supervisor’s supervisory philosophy, including whether it emphasizes independent research ability and academic norms.	1 = very dissatisfied; 2 = dissatisfied; 3 = neutral; 4 = satisfied; 5 = very satisfied
A4	PSG	Timeliness of guidance	您对导师指导及时性的满意程度，如遇到研究问题时能否及时反馈、答疑和指导。	Your satisfaction with the timeliness of your supervisor’s guidance, including timely feedback, responses to questions, and guidance when research problems arise.	1 = very dissatisfied; 2 = dissatisfied; 3 = neutral; 4 = satisfied; 5 = very satisfied
A5	PSG	Resource provision	您对导师提供资源支持的满意程度，如经费、设备、数据、平台、学术交流机会等。	Your satisfaction with the research resources provided by your supervisor, such as funding, equipment, data, platforms, and academic exchange opportunities.	1 = very dissatisfied; 2 = dissatisfied; 3 = neutral; 4 = satisfied; 5 = very satisfied
B1	PGAMS	Institutional implementation	您对学院／学校执行研究生培养相关制度，如开题、中期、答辩、评奖评优等的满意程度。	Your satisfaction with the college/university implementation of graduate-training requirements, such as proposal defense, mid-term assessment, thesis defense, awards, and evaluations.	1 = very dissatisfied; 2 = dissatisfied; 3 = neutral; 4 = satisfied; 5 = very satisfied
B2	PGAMS	Academic process management	您对学院／学校学业管理规范性、事务办理流程清晰度和服务的满意程度。	Your satisfaction with the standardization of academic management, the clarity of administrative procedures, and related services provided by the college/university.	1 = very dissatisfied; 2 = dissatisfied; 3 = neutral; 4 = satisfied; 5 = very satisfied
B3	PGAMS	Life-related support	您对学院／学校提供的生活保障，如住宿、补助、医疗、学习环境等的满意程度。	Your satisfaction with life-related support provided by the college/university, such as accommodation, financial assistance, medical services, and the learning environment.	1 = very dissatisfied; 2 = dissatisfied; 3 = neutral; 4 = satisfied; 5 = very satisfied
B4	PGAMS	Psychological care	您对学院／学校或培养单位关注研究生心理状态并提供关怀与支持的满意程度。	Your satisfaction with the attention, care, and support provided by the college/university or training unit for graduate students’ psychological state.	1 = very dissatisfied; 2 = dissatisfied; 3 = neutral; 4 = satisfied; 5 = very satisfied
C1	RDS	Research engagement	我目前能积极投入科研工作，保持较高的科研投入状态。	I am currently able to engage actively in research and maintain a relatively high level of research involvement.	1 = strongly disagree; 2 = disagree; 3 = neutral; 4 = agree; 5 = strongly agree
C2	RDS	Perceived research competence	我具备完成本专业研究所需的科研能	I possess the research abilities needed to conduct research in my discipline, such as literature reading,	1 = strongly disagree; 2 = disagree; 3 = neutral; 4 =

Item	Construct	Aspect	Original Chinese wording	English translation	Response scale
			力，如文献阅读、研究方法、论文写作等。	research methods, and academic writing.	agree; 5 = strongly agree
C3	RDS	Research output	我目前取得了较好的科研产出，如论文、专利、项目、报告、竞赛等。	I have currently achieved research outputs such as papers, patents, projects, reports, or competition outcomes.	1 = strongly disagree; 2 = disagree; 3 = neutral; 4 = agree; 5 = strongly agree
C4	RDS	Research–life balance	我能较好平衡科研工作与个人生活，保持身心健康。	I am able to balance research work and personal life while maintaining psychological and physical well-being.	1 = strongly disagree; 2 = disagree; 3 = neutral; 4 = agree; 5 = strongly agree

Note. PSG = perceived supervisor guidance; PGAMS = perceived graduate affairs management support; RDS = self-reported research development status. The original instrument was administered entirely in Chinese. The English translations are provided for reporting purposes only and were not used for data collection. The original response instructions used the paired anchors “strongly disagree/very dissatisfied” to “strongly agree/very satisfied”; Parts A and B were answered as satisfaction ratings, whereas Part C was answered as agreement ratings.

Supplementary Table S2. Item-Level Results of the Principal Component Analysis

Item	Expected construct	PSG component	PGAMS component	RDS component	Highest cross-loading	Communality
A1	PSG	0.773	0.055	0.098	0.098	0.610
A2	PSG	0.772	0.164	0.076	0.164	0.629
A3	PSG	0.774	0.174	0.165	0.174	0.657
A4	PSG	0.770	0.191	0.107	0.191	0.640
A5	PSG	0.745	0.202	0.105	0.202	0.607
B1	PGAMS	0.084	0.780	0.214	0.214	0.661
B2	PGAMS	0.207	0.794	0.132	0.207	0.690
B3	PGAMS	0.186	0.787	0.168	0.186	0.682
B4	PGAMS	0.160	0.796	0.162	0.162	0.686
C1	RDS	0.102	0.204	0.775	0.204	0.652
C2	RDS	0.091	0.160	0.814	0.160	0.697
C3	RDS	0.091	0.126	0.829	0.126	0.711
C4	RDS	0.159	0.164	0.782	0.164	0.664
**Rotated SS loadings**		3.103	2.738	2.744		
**Variance explained (%)**		23.872	21.063	21.108		
**Cumulative variance (%)**		23.872	44.935	66.043		

Note. N = 378. Principal component extraction with Varimax rotation was performed on the 13 items. KMO = 0.885; Bartlett's test of sphericity, $\chi^2(78) = 2066.899$, $p < 0.001$. Three components had eigenvalues > 1 before rotation (5.004, 2.050, and 1.531), jointly explaining 66.04% of the variance. Primary rotated loadings ranged from 0.745 to 0.829; the largest cross-loading was 0.214.

Supplementary Table S3. Confirmatory Factor Analysis, Reliability, and Construct Validity

Panel A. Item-level standardized CFA loadings

Construct	Item	Standardized factor loading
PSG	A1	0.687
PSG	A2	0.728
PSG	A3	0.764
PSG	A4	0.745
PSG	A5	0.722
PGAMS	B1	0.726
PGAMS	B2	0.767
PGAMS	B3	0.766
PGAMS	B4	0.762
RDS	C1	0.735
RDS	C2	0.771
RDS	C3	0.779
RDS	C4	0.743

Panel B. Internal consistency and convergent validity

Construct	No. of items	Cronbach's α	Composite reliability (CR)	Average variance extracted (AVE)
PSG	5	0.850	0.850	0.532
PGAMS	4	0.841	0.842	0.570
RDS	4	0.843	0.843	0.573

Panel C. Heterotrait–monotrait (HTMT) ratios

Construct	PSG	PGAMS	RDS
PSG	—	0.481	0.360
PGAMS		—	0.493
RDS			—

Panel D. Three-factor versus one-factor CFA model fit

Model	χ^2	df	p	CFI	TLI	RMSEA	SRMR
Three-factor	60.35	62	.536	1.000	1.000	0.000	0.028
One-factor	855.03	65	<.001	0.608	0.530	0.180	0.133

Note. N = 378. The three-factor CFA specified correlated PSG, PGAMS, and RDS factors. The unbounded TLI estimate was slightly above 1 and is reported at the conventional upper bound of 1.000. Harman's single-factor test indicated that the first unrotated component accounted for 38.49% of total variance. The one-factor CFA fit substantially worse than the three-factor model; these diagnostics do not eliminate the possibility of common method variance but do not indicate a dominant single common factor.

Supplementary Table S4. Illustrative Open Coding and Source Traceability From the Qualitative Interviews

Participant	Participanttype	Abridged Chinese source excerpt/English translation	Initial code or concept	Focused category
T01	Supervisor	中文：组会上每个学生都要汇报自己的进展……至少要讲清楚这周做了什么、遇到什么问题、下周准备做什么。<br>English: In group meetings, every student reports progress and must explain what was done that week, what problems were encountered, and what is planned for the next week.	Weekly progress reporting; progress monitoring	Research process training
T02	Supervisor	中文：文献汇报……不只是翻译论文，而是要讲清楚这篇文章解决了什么问题、方法创新在哪里、实验设计是否合理、对我们自己的课题有什么启发。<br>English: Literature presentations should not merely translate papers; students must explain the problem addressed, methodological innovation, the adequacy of the experimental design, and implications for their own projects.	Analytical reading; methodological learning	Research process training
S01	Graduate student	中文：导师帮我把现阶段任务分清楚：第一是完成课程基础，第二是读懂经典模型，第三是复现一个可运行的 baseline，第四是逐步比较几个候选题目。这样我就没有一开始那么乱了。<br>English: My supervisor separated the current tasks into building the course foundation, understanding classic models, reproducing a workable baseline, and gradually comparing candidate topics. This made the initial stage much less confusing.	Task decomposition; research-entry support	Supervisor academic guidance
S02	Graduate student	中文：知道自己应该努力，但不知道努力的路径是否正确。<br>English: I knew that I should make an effort, but I did not know whether the path of my effort was correct.	Uncertain research pathway; unclear research-entry direction	Support gaps
S04	Graduate student	中文：导师经常说，实验失败并不可怕，可怕的是不知道为什么失败，也没有记录失败过程。<br>English: My supervisor often says that experimental failure is not frightening; what is frightening is not knowing why it failed and not recording the failure process.	Failure analysis; methodological reflection	Supervisor academic guidance
GMA01	Graduate affairs administrator	中文：从研究生入学以后，培养计划制定、课程选课、学分审核、培养方案执行、开题安排、中期考核……论文送审、答辩资格审核、学位申请材料整理、毕业材料归档，这些基本都和我有关。<br>English: From enrollment onward, training-plan formulation, course selection, credit review, implementation of the training plan, proposal defense, mid-term assessment, thesis review, defense qualification, degree-application materials, and graduation archiving all fall within my work.	Continuous milestone management	Training process management
GMA03	Graduate affairs administrator	中文：比如学生开题延期、中期考核不通过、论文长期没有初稿、导师反馈学生不主动、学生心理压力较大、导学沟通不畅、延期风险较高，这些都需要学院层面提前识别和介入。<br>English: Delayed proposal defense, failed mid-term assessment, prolonged absence of a thesis draft, low student initiative, psychological pressure, poor supervisor–student communication, and graduation-delay risk all need early identification and intervention at the college level.	Early risk identification; proactive intervention	Training process management
S03	Graduate student	中文：管理老师把流程解释清楚之后，很多未知就变成已知。虽然事情还是要做，但心理上没那么慌。	Procedural clarification; reduced uncertainty	Training process management

Participant	Participanttype	Abridged Chinese source excerpt/English translation	Initial code or concept	Focused category
GMA04	Graduate affairs administrator	English: After the administrator explained the process clearly, many unknowns became known. The tasks still had to be done, but psychologically I felt less panicked.<br>中文：我认为心理支持、导学关系支持和组织文化支持非常关键。制度和流程是基础，但研究生能不能持续投入科研，很大程度上取决于他是否有稳定的心理状态，是否和导师保持有效沟通，是否在学院和课题组中感到被支持。<br>English: Psychological support, support for the supervisor–student relationship, and organizational culture are critical. Rules and procedures are foundational, but sustained research engagement also depends on psychological stability, effective communication with the supervisor, and feeling supported in the college and research group.	Psychological and relational support; organizational climate	Student support services
S17	Graduate student	中文：主要是导师引导和管理老师的心理支持共同作用。导师帮我把审稿意见具体化，管理老师和辅导员那边则提醒我们……不要把一次评价等同于自我价值。<br>English: The change mainly came from the combination of supervisor guidance and psychological support from administrative staff. My supervisor helped make the reviewers' comments concrete, while administrative staff reminded us not to equate one evaluation with our self-worth.	Post-rejection support; psychological stabilization	Research pressure and development experiences
S18	Graduate student	中文：在我的经历中，桥梁作用最明显……管理老师不直接替我决定题目，也不评价导师指导好不好，但她帮助我把问题表达出来，提醒我和导师沟通，也告诉我培养流程上怎么处理换题。<br>English: In my experience, the bridging role was most evident. The administrator did not decide my topic or evaluate my supervisor, but helped me articulate the problem, communicate with my supervisor, and understand how to handle the topic change within the training process.	Communication bridge; procedural coordination	Cross-category analytic integration
T01	Supervisor	中文：导师是学术培养的第一责任人，主要负责研究方向、科研训练、论文质量和学术规范……管理者是制度支持和发展支持的提供者，负责流程管理、资源协调、风险识别和服务保障。<br>English: Supervisors bear primary responsibility for academic training, including research direction, research training, thesis quality, and academic norms; administrators provide institutional and developmental support through process management, resource coordination, risk identification, and service assurance.	Role differentiation; complementary responsibilities	Cross-category analytic integration
T03	Supervisor	中文：导师可以关心，但不是所有问题都能由导师解决。<br>English: Supervisors can provide care, but not every problem can be solved by the supervisor.	Referral need; support boundary	Cross-category analytic integration

Note. Coding and theme development were conducted in Chinese. The Chinese text in this table is an abridged source excerpt from the de-identified interview materials; the English text is a reporting translation. Excerpts are illustrative rather than exhaustive and are not used as frequency counts. Participant identifiers follow the manuscript: S = graduate student; T = supervisor; GMA = graduate affairs administrator.

Supplementary Table S5. Development From Initial Codes to Focused Categories and Core Themes

Illustrative initial codes and supporting participant IDs	Focused category / analytic integration	Core theme
Topic-selection support; clarification of research directions; methodological training; experimental guidance; thesis revision; resource provision. Illustrative sources: T01, T02, T03, T04, T05, S01, S04, S18.	Supervisor academic guidance	Academic entry and research training through supervisor guidance
Research group meetings; one-to-one discussions; project-task breakdown; stage-based feedback; peer collaboration; experiment/analysis review. Illustrative sources: T01, T02, S01, S04, S08.	Research process training	Academic entry and research training through supervisor guidance
Training-plan interpretation; proposal-defense reminders; mid-term assessment; thesis review and defense; graduation review; risk monitoring. Illustrative sources: GMA01, GMA03, GMA06, S03, S11, S18.	Training process management	Procedural assurance and risk identification through graduate affairs management support
Scholarship and financial aid support; life-related support; psychological care; employment support; resource referral. Illustrative sources: GMA04, GMA06, T01, S17.	Student support services	Procedural assurance and risk identification through graduate affairs management support
Delayed or insufficient feedback; unclear requirements or procedures; unclear help-seeking channels; communication avoidance; information asymmetry. Illustrative sources: S02, S03, S07, GMA03.	Support gaps	Uncertainty and pressure under support gaps
Changes in research engagement; competence development; experimental frustration; research-output pressure; graduation anxiety; research–life imbalance. Illustrative sources: S04, S05, S11, S14, S17.	Research pressure and development experiences	Uncertainty and pressure under support gaps
Comparative analysis of academic-judgment codes and institutional/process-support codes; role differentiation; referral and coordination; non-substitution of academic judgment. Illustrative sources: T01, T03, GMA03, GMA04, GMA06, S18.	Cross-category analytic integration	Institutional support functions and boundaries

Note. Participant IDs in the first column indicate illustrative supporting sources and are not frequency counts. The first six rows summarize the progression from initial codes to focused categories. The final row shows cross-category integration used to develop the institutional support functions and boundaries theme.

Supplementary Table S6. Detailed Diagnostics of the Four- and Five-Profile Solutions

Panel A. Solution-level diagnostics

Solution	AIC	BIC	aBIC	Entropy	Hard-class sizes	Minimum class	AvePP range	Model status
4-profile	8672.5	8967.6	8729.6	0.855	129 / 94 / 103 / 52	13.8%	0.912–0.939	Selected
5-profile	7865.2	8235.1	7936.9	0.868	78 / 41 / 80 / 82 / 97	10.8%	0.870–0.982	Near-singular / inadmissible

Panel B. Class-level diagnostics for the selected four-profile solution

Profile	n	%	AvePP	Mean PSG	Mean PGAMS	Minimum class-specific indicator variance	Status
Fully supported	129	34.1	0.912	4.252	4.191	0.383	Admissible
Management-support-dominant	94	24.9	0.913	2.892	3.966	0.444	Admissible
Supervisor-support-dominant	103	27.2	0.934	3.555	2.836	0.469	Admissible
Insufficiently supported	52	13.8	0.939	2.264	2.503	0.148	Admissible

Panel C. Class-level diagnostics for the five-profile solution

Profile	n	%	AvePP	Mean PSG	Mean PGAMS	Minimum class-specific indicator variance	Status
Profile 1	78	20.6	0.982	2.431	3.012	1.0×10^{-6}	Variance collapse at A2; inadmissible
Profile 2	41	10.8	0.917	4.573	4.628	0.185	Part of inadmissible solution
Profile 3	80	21.2	0.930	3.304	2.672	0.431	Part of inadmissible solution
Profile 4	82	21.7	0.893	3.157	4.093	0.418	Part of inadmissible solution
Profile 5	97	25.7	0.870	4.123	3.678	0.363	Part of inadmissible solution

Note. AIC = Akaike information criterion; BIC = Bayesian information criterion; aBIC = sample-size-adjusted BIC; AvePP = average posterior probability; PSG = perceived supervisor guidance; PGAMS = perceived graduate affairs management support. Although the five-profile solution had lower information criteria, at least one class-specific indicator variance reached the numerical lower bound (approximately 1×10^{-6}), indicating variance collapse and a near-singular boundary solution. The four-profile solution was therefore retained.

Supplementary Figure S1. Indicator Profiles for the Four- and Five-Profile Solutions

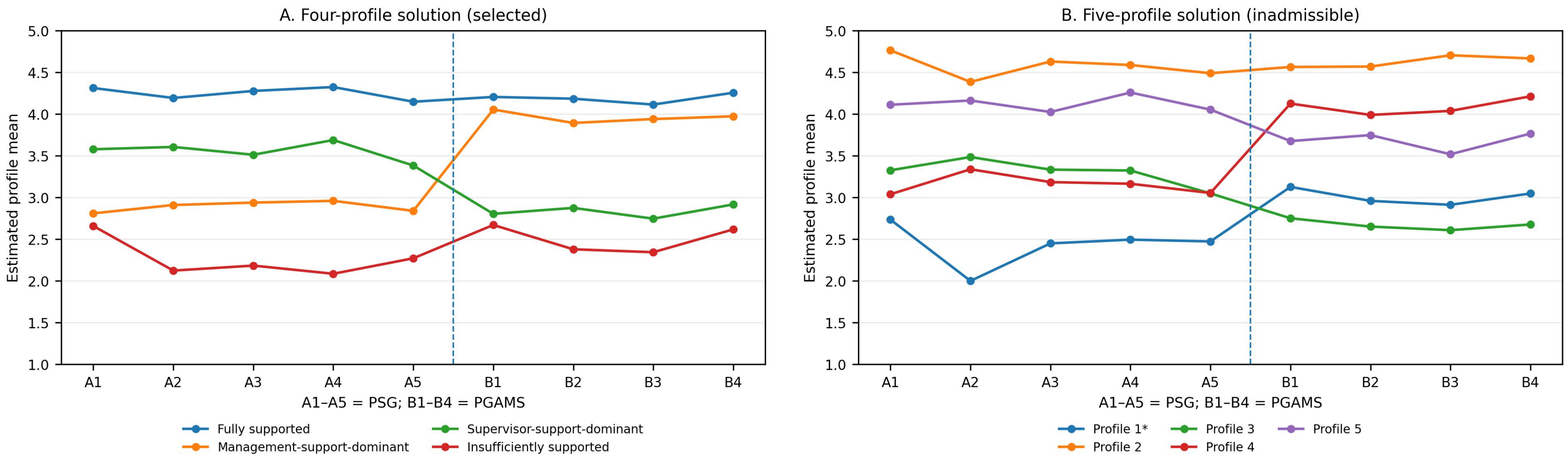

Note. Panel A shows the selected four-profile solution. Panel B shows the five-profile solution that was not retained because it was near-singular/inadmissible. The asterisk identifies Profile 1, in which the class-specific variance for A2 converged to approximately 1×10^{-6}. A1–A5 are perceived supervisor guidance indicators and B1–B4 are perceived graduate affairs management support indicators. The y-axis uses the original 1–5 response scale. Profile numbers in Panel B are descriptive only and should not be interpreted as substantive labels.
